# Does the phubbing scale measure the same construct across sexes? Evidence from a Nigerian sample

**DOI:** 10.3389/fpsyt.2026.1820789

**Published:** 2026-05-22

**Authors:** Olga Malas, Dayo Omotoso, Mirko Duradoni, Maciej Załuski

**Affiliations:** 1 Department of Psychology, Sociology and Social Work, University of Lleida, Lleida, Spain; 2 Department of Human Anatomy, Redeemer’s University, Ede, Osun State, Nigeria; 3 Department of Human and Social Sciences, Mercatorum University, Rome, Italy; 4 Chair of Bioethics and Health Psychology, Faculty of Health Sciences, Jagiellonian University Medical College, Krakow, Poland

**Keywords:** digital life balance, invariance, phubbing, phubbing scale, psychometric validation, technological addictions

## Abstract

**Introduction:**

Phubbing is associated with excessive use of digital technologies, and the Phubbing Scale (PS) is widely used to measure it. The PS may be influenced by cultural variations, so it is advisable to validate it before applying it in a new cultural context. Phubbing has emerged as a construct of interest in Nigeria, yet no validation studies for the PS have been conducted in this population.

**Methods:**

To address this gap, the PS, along with scales measuring technological addictions and digital life balance, was administered to 396 Nigerian university students (18 to 25 years old; 80.6% female). Confirmatory Factor Analysis was used to test two 10-item models and one 8-item model. The best-fitting model was subsequently tested. Internal and external validity, and measurement invariance across sexes were assessed.

**Results:**

The 8-item correlated two-factor model (PS-8) showed the best results. For PS-8, internal and external validity, and strict measurement invariance across sexes were supported based on global fit indices. However, parameter estimates suggested potential sex-related differences in the construct. In females, factor loadings and the correlation between factors were adequate, whereas in males several items of Factor 2 showed low loadings, and the correlation between factors was negligible.

**Conclusion:**

In conclusion, the PS-8 appears suitable for measuring phubbing in this population, although potential sex-related differences should be considered. These differences may stem from psychometric limitations in the Smartphone Obsession factor items and/or from variations in how the PS construct manifests across sexes, which could affect interventions or comparative studies. Considering the limitations of the study, future research will be necessary to corroborate these findings.

## 1 Introduction

So-called smartphones facilitate communication between people and access to information, which explains why they have become an essential part of our daily lives (1). Despite their benefits, excessive use can lead to problems of addiction or dependency on communication through virtual environments, which may affect the social and emotional life of those.

A particular feature of smartphones is that they can both bring us closer to others by facilitating communication and distance or disconnect us from those around us (2). In this context, the phenomenon of *phubbing* has emerged, which consists of ignoring the people present in order to pay attention to one’s phone (1, 3). The term *phubbing* arises from the combination of the words *phone* and *snubbing* and refers to the behaviour of continuously checking one’s phone during a face-to-face conversation, thereby ignoring the interlocutor (1).

This behaviour prioritises smartphone use over interpersonal interactions, affecting the quality of social relationships (1, 4, 5) and life satisfaction (3). It has also been associated with boredom (6, 7), loneliness, low self-esteem (8), worry, anxiety (1), social anxiety (9, 10) stress (11), depression (3, 11), negative affectivity (12) and technological addictions (3).

In Nigeria, the majority of university students use the Internet and smartphones for both academic work and social purposes (13). Reported rates of problematic smartphone use in this population vary widely, ranging from 17.34% to 88% (13–16). These data highlight the high prevalence of intensive and potentially dysfunctional smartphone use among Nigerian youth, creating a context particularly conducive to the emergence of behaviours such as phubbing. Despite this, following a review of the literature, no studies on phubbing among Nigerian university students were found. Only one study has been identified, conducted by Aloh et al. (17), which focused on couples with children, and reported daily phubbing behaviour along with associated relational consequences, including increased marital conflict and tension (37.5%), reduced marital satisfaction (15%), and even relationship dissolution (7.5%). It is noteworthy that in Nigeria, the strong cultural emphasis on family unity and relational cohesion may amplify the negative effects of this behaviour (17). Overall, the combination of a high prevalence of problematic smartphone use, the scarcity of research on phubbing, and evidence of significant relational consequences in families with children underscores the urgent need to study phubbing in Nigerian populations. These findings are particularly relevant among university students, as they represent a key group for the prevention of long-term dysfunctional relational patterns.

Several instruments have been developed to measure phubbing (e.g.: 1, 3, 18). One of the most widely used is the Phubbing Scale (PS) developed by Karadağ et al. (1). The scale consists of 10 items distributed across two theoretical dimensions: communication interference (items 1 to 5) and smartphone obsession (items 6 to 10). Although conceptually phubbing focuses on social interference, the authors argue that this behaviour does not occur in isolation but is driven by a strong need or dependence on the smartphone.

According to the authors, without this underlying motivation, social interference would not consistently occur. Notably, the factor analysis conducted by Karadağ et al. (1) redistributed the items into the final dimensions: communication interference comprised items 1, 2, 3, 4, and 10, while smartphone obsession comprised items 5, 6, 7, 8, and 9. This demonstrates that some items theoretically intended for one dimension were more strongly correlated with items of the other. Thus, the empirical factorial structure represents how the items naturally cluster according to their statistical behaviour, differing from the initial theoretical intention.

This scale has been validated in a considerable number of countries and cultures, showing a high variability in results. Veysuei et al. (19) successfully reproduced the scale reported by Karadağ et al. (1) in a young adult Pakistani sample. However, this has not been the case in other validations published to date. For example, in a sample of Spanish adults (20) and another of Spanish adolescents and young adults (21), the best fit indices were obtained for the original two-factor theoretical structure (F1: 1 to 5; F2: 6 to 10), with scalar invariance reported across sexes. The same result was found by Zamani et al. (22) in Iranian university students. In contrast, Mendes et al. (23), in Portuguese adults, reported a 10-item scale distributed across two correlated factors with a different allocation of items between factors. Similarly, Santos et al. (24), in Brazilian adults, obtained the best fit indices for a 9-item scale (F1: 1 to 4; F2: 6 to 10). Notably, Błachnio et al. (25), in an adult population sample from 20 countries (Belarus, Brazil, China, Croatia, Ecuador, India, Israel, Italy, Netherlands, Pakistan, Poland, Portugal, Serbia, Slovakia, Slovenia, Spain, Turkey, UK, Ukraine, and USA), reported that an 8-item scale, distributed across two correlated factors (F1: 1 to 4; F2: 6 to 9), presented the best fit indices, as well as scalar invariance across sexes and metric invariance across countries. Lin et al. (26), in adults from Bangladesh, Iran, and Pakistan, reported similar results for the PS-8 proposed by Błachnio et al. (25), both in terms of structure and invariance. This same structure and invariance result has also been reported by García-Castro et al. (27) for a sample of Portuguese adults, and by Kim et al. (28) in a sample of Thai adolescents.

Another characteristic observed in some of these studies is the presence of RMSEA values above.080 (e.g., 19, 21, 25, 28); Cronbach’s alpha values below.80 for one or the two factors (21, 25, 27, 28); or AVE values below.50 (23). While factor correlations are commonly above 0.60 (23), some studies report correlations as low as.51 (28) or even.24 (22).

The considerable variability observed in the results of different validations of the PS across countries and cultures highlights that the factorial structure and psychometric properties of the scale may not be universal. Differences in item allocation, fit indices, internal consistency, and inter-factor correlations across studies and populations, suggest that the way individuals’ experiences of phubbing may be influenced by cultural and contextual factors. These findings support the notion that culture significantly influences how phubbing is perceived and enacted (29).

Due to the growing interest in the study of phubbing and the importance of having reliable tools to measure it, it is necessary to adapt existing instruments to different cultural contexts, such as Nigeria. This will allow research on phubbing to progress at a national level and to assess whether existing models fit adequately or need to be adapted to the new cultural context.

Therefore, considering that an English version of the Phubbing Scale (PS) already exists, the main objective of this study is to examine its psychometric properties in this population. Moreover, it is essential to assess measurement invariance across sexes, and its potential influence on the manifestation of phubbing and the interpretation of items. Establishing this invariance ensures that comparisons between male and female are valid and unbiased, allowing for the reliable use of the scale in research and interventions (30) within the Nigerian context.

## 2 Methods

### 2.1 Procedure

Data were collected in September 2024 using a non-probability convenience sampling method. Participants were invited via university mailing lists linked to their faculties with an explanation of the study’s aims, informed consent form, and a link to the online questionnaire (Microsoft Forms). The system prevented submission of incomplete responses, ensuring a complete dataset. Eligible participants were university students aged 18 to 25 who were enrolled at the time of data collection.

### 2.2 Participants

The final sample comprised 396 students, of whom 19.4% were male and 80.6% were female. The sample size was considered adequate according to established methodological recommendations, which suggest at least ten participants per variable (31) and a minimum of 300 participants to ensure stable factor-analytic results (32).

All participants provided written informed consent prior to participation. The consent form explained the study’s objectives, the voluntary nature of participation, the absence of incentives, and the measures taken to ensure confidentiality and anonymity, as well as the option to withdraw at any time. The study protocol received ethical approval from the Research Ethics Committee of the Directorate for Research, Innovation, and Partnerships at Redeemer’s University, Nigeria, on 15 April 2024 (reference number RUN/REC/2024/115).

### 2.3 Instruments

#### 2.3.1 Phubbing scale

The original 10-item Phubbing Scale (PS-10; 1) was analysed. In addition, the alternative 10-item version validated by Blanca and Bendayan (20) and Barbed-Castrejón et al. (21) was also tested. Furthermore, the 8-item version (PS-8) validated by Błachnio et al. (25)Lin et al. (26), among others, was tested. Examples of items include, for the Communication Interference factor (F1), “People complain about me using my mobile phone”, and for the Smartphone Obsession factor (F2), “I feel incomplete without my mobile phone”. Items are rated on a 5-point Likert scale, ranging from 1 (never) to 5 (always). Each factor is scored from 5 to 25 (PS-10) or from 4 to 20 (PS-8), with total scores ranging from 10 to 50 and from 8 to 40, respectively. Higher scores indicate greater communication disruption and stronger mobile phone involvement.

#### 2.3.2 Smartphone application-based addiction scale

The Smartphone Application-Based Addiction Scale (SABAS; 33) consists of 6 items rated on a 6-point Likert scale, ranging from 1 (strongly disagree) to 6 (strongly agree). Smartphone addiction behaviour is assessed using a unidimensional model. Participants report the frequency of specific behaviours (e.g., “I find that my smartphone use interferes with my daily responsibilities or social interactions.”). The scale is scored from 6 to 36, with higher scores indicating a greater risk of developing an addiction to smartphone applications.

#### 2.3.3 Bergen social media addiction scale

The Bergen Social Media Addiction Scale (BSMAS; 34) consists of a 6-item unidimensional scale used to assess social media addiction behaviour. Items are rated on a 5-point Likert scale, ranging from 1 (almost never) to 5 (almost always). Participants report their experiences with social media over the past year (e.g., “During the past year, how often have you felt nervous or upset when you were not allowed to use social media?”). The scale is scored from 6 to 30, with higher scores indicating a greater risk of social media addiction.

#### 2.3.4 Game addiction scale

The Game Addiction Scale (GAS-7; 35) consists of 7 items rated on a 5-point Likert scale, ranging from 1 (never) to 5 (very often). Gaming addiction behaviour is assessed using a unidimensional model based on the DSM-5 criteria for pathological gaming. Participants report the frequency of specific behaviours experienced over the past six months (e.g., “Did you feel bad when unable to play?”). The scale is scored from 7 to 35, with higher scores indicating a greater risk of gaming addiction.

#### 2.3.5 The digital life balance scale

The Digital Life Balance Scale (DLB-S; 36) consists of 4 items rated on a 7-point Likert scale, ranging from 1 (strongly disagree) to 7 (strongly agree). Digital life balance is assessed using a unidimensional model. Participants report the extent to which they achieve balance in their use of digital technologies (e.g., “Overall, I believe that my online and offline life are balanced”). The scale is scored from 4 to 28, with higher scores reflecting a better balance between digital and offline life.

Internal consistency results (coefficient α and ω) for these scales, based on data collected in the present study, is reported in **Table 1**.

**Table 1:** Items analysis and internal and external validity for PS-8 (n=396).

Item	Mean	*SD*		Skewness		Kurtosis	Coefficient α (if item dropped)		Item-rest correlation	
PS-1	2.778	2.778		-0.044		-0.980	.763		.572	
PS-2	2.379	2.379		0.515		-0.190	.754		.653	
PS-3	2.126	2.126		0.856		0.446	.772		.520	
PS-4	2.278	2.278		0.541		-0.162	.757		.630	
PS-6	3.096	3.096		-0.320		-1.000	.809		.302	
PS-7	3.588	3.588		-0.974		0.703	.777		.487	
PS-8	2.740	2.740		0.043		-1.038	.783		.452	
PS-9	2.604	2.604		0.237		-0.955	.775		.504	
Scales	Mean	*SD*		Skewness		Kurtosis	Coefficient α	Coefficient ω	AVE	HTMT
PS total	21.588	5.517		-0.243		0.491	.797	.777	--	--
PS-F1	9.561	3.311		0.233		0.011	.838	.841	.576	.605
PS-F2	12.028	3.174		-0.446		0.057	.652	.667	.349	
SABAS total	19.412	5.800		-0.159		-0.286	.787	.792	--	--
BSMAS total	14.934	4.546		0.309		-0.122	.807	.808	--	--
GAS total	11.492	4.934		1.167		1.059	.878	.880	--	--
DLB total	18.235	5.943		-0.412		-0.808	.880	.881	--	--
Pearson Correlations		1		2	3	4	5	6	7	
1. PS total		—								
2. PS-F1		.857		—						
3. PS-F2		.844		.447	—					
4. SABAS total		.582		.503	.487	—				
5. BSMAS total		.497		.454	.390	.649	—			
6. GAS total		.312		.289	.240	.233	.285	—		
7. DLB total		-.296		-.278	-.224	-.281	-.480	-.172	—	

*SD*, Standard deviation. All correlation were significant at the 0.01 level (two-tailed).

### 2.4 Statistical analysis

All analyses were conducted using JASP software (version 0.95.1.0) with the Lavaan add-on.

To examine the factorial structure of the PS, confirmatory factor analysis (CFA) was performed using maximum likelihood (ML) estimation. In order to select the best-fitting model, the unidimensional model (M1 = items 1 to 10), the original model (M2 = F1: items 1, 2, 3, 4, 10; F2: items 5, 6, 7, 8, 9), the alternative 10-item model (M3 = F1: items 1 to 5; F2: items 6 to 10), and the 8-item model (M4 = F1: items 1 to 4; F2: items 6 to 9) were evaluated. Model fit was assessed using a combination of absolute and incremental fit indices, including the chi-square to degrees of freedom ratio (χ²/df), the Comparative Fit Index (CFI), the Tucker–Lewis Index (TLI), the Root Mean Square Error of Approximation (RMSEA) with 90% confidence intervals, and the Standardised Root Mean Square Residual (SRMR). According to recommended cut-off values, a χ²/df ≤ 3 was considered indicative of good fit, and values ≤ 5 acceptable; CFI and TLI values ≥ 0.95 indicated excellent fit, whereas values > 0.90 were considered acceptable; RMSEA values < 0.06 reflected good fit, values < 0.08 acceptable fit, and values < 0.1 poor fit; and SRMR values < 0.08 were interpreted as adequate (37).

For the best-fitting scale, item analysis was conducted. Descriptive statistics (means, standard deviations, skewness, and kurtosis) were computed. Also, the Cronbach’s alpha if item deleted and corrected item–total correlations were calculated to evaluate the contribution of each item to the internal consistency of the scale.

Reliability, together with internal and external validity, was subsequently assessed. Cronbach’s alpha (α) and McDonald’s omega (ω) coefficients were calculated as measures of reliability. For the α coefficient, values >.90 were considered excellent, >.70 good, and >.60 acceptable (38). The α and ω values differed only in cases of small sample sizes (50 and 100), low factor loadings (.3 and.4), and when the scale contains 5-tems or fewer (39). Considering that the PS factors consist of 4–5 items, both coefficients were calculated. Following, convergent validity was examined using the Average Variance Extracted (AVE), with values of 0.50 or higher indicating satisfactory convergence; whereas discriminant validity between factors was assessed using the Heterotrait–Monotrait ratio (HTMT) with values of 0.85 or less indicating satisfactory discriminant validity (40). To examine their external validity, correlation coefficients were calculated between PS scores (total and subscales) and measures of SABAS, BSMAS, GAS, and DLBS.

Finally, measurement equivalence across males and females was evaluated using multigroup confirmatory factor analysis (MGCFA). Invariance testing was conducted progressively following a hierarchical approach: first, configural invariance was examined to determine whether the basic factorial structure was similar across both groups; next, metric invariance was tested by constraining factor loadings to be equal; then, scalar invariance was assessed by imposing equality constraints on item intercepts; and finally, strict invariance was evaluated by constraining residual variances to be equal (30, 41). Factor loadings, factor correlations, and other model parameters were analysed, and the resulting models were compared using changes in fit indices (ΔCFI ≤ 0.01 and ΔRMSEA ≤ 0.015), with invariance considered to be supported when these criteria were met (42).

## 3 Results

### 3.1 Confirmatory factor analysis

The CFA results are presented in **Table 2**. The first step involved evaluating the 10-item unidimensional model (Model 1) to examine whether the scale could be considered essentially unidimensional. However, this model showed a clearly inadequate fit. These results suggest that the items do not form a single homogeneous construct, but rather reflect more than one underlying dimension. Subsequently, Models 2 (original validated version) and 3 (theoretical version) were evaluated, both consisting of two correlated five-item factors. Although these models showed slight improvements compared with the unidimensional solution, their fit indices were still insufficient.

**Table 2:** Confirmatory factor analysis (n=396).

	χ²/df	p	CFI	TLI	RMSEA	RMSEA IC90%	SRMR
Model 1	8.58	<.001	.808	.754	.138	.124,.153	.082
Model 2	4.41	<.001	.916	.889	.093	.078,.108	.068
Model 3	5.19	<.001	.897	.864	.103	.080,.118	.075
Model 4	3.85	<.001	.949	.925	.085	.065,.106	.051

χ²/df, chi-square/degrees of freedom ratio; CFI, Comparative Fit Index; TLI, Tucker–Lewis Index; RMSEA, Root Mean Square Error of Approximation; CI, confidence interval; SRMR, Standardised Root Mean Square Residual; Model 1: PS-10-Unifactorial (items 1,2,3,4,5,6,7,8,9,10); Model 2: PS-10-Two correlated factors (F1: items 1,2,3,4,5; F2: items 6,7,8,9,10); Model 3: PS-10-Two correlated factors (F1: items 1,2,3,4,10; F2: items 5,6,7,8,9); Model 4: PS-8-Two correlated factors (F1:1,2,3,4; F2: 6,7,8,9).

In contrast, Model 4, which retained eight items organised into two correlated factors (items 1–4 and 6–9), achieved satisfactory fit indices. In addition to an overall acceptable fit, the RMSEA was somewhat elevated, whereas the CFI/TLI and SRMR fell within recommended ranges (CFI/TLI >.90; RMSEA = .085; SRMR = .051). Given that RMSEA tends to penalize models with a small number of degrees of freedom, interpretative priority should be given to CFI, TLI, and SRMR.

**Figure 1:**
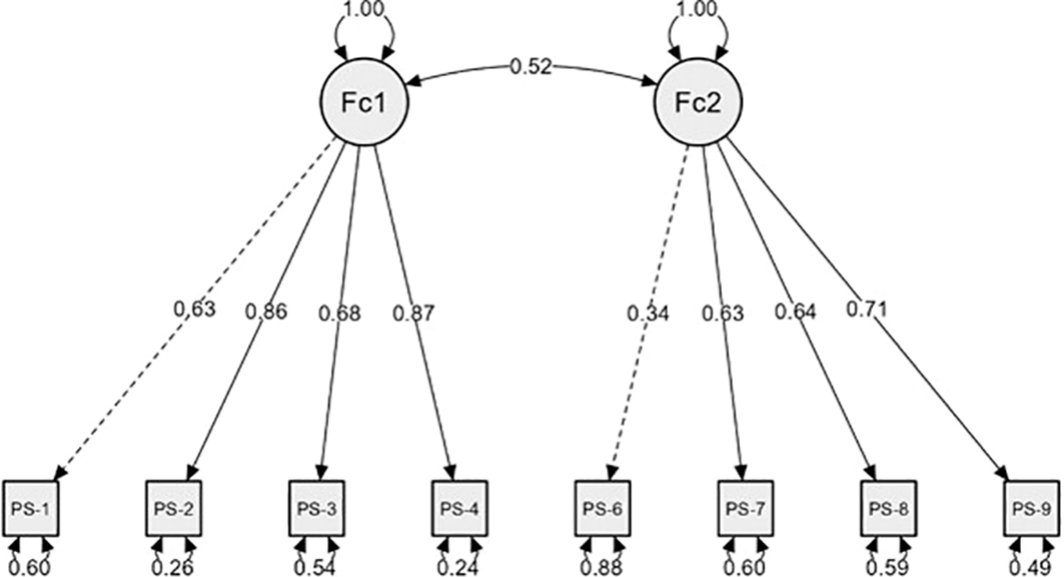
Model plot for PS-8 structural model (n=396).

**Figure 1** shows the structural model for the 8-item PS. Inspection of the standardised factor loadings indicated that all items loaded strongly on their intended factors (>.60), with the exception of item 6, which showed a notably lower loading (.34). This finding suggests a weaker association between item 6 and its corresponding latent factor, indicating that this item may capture a smaller proportion of the underlying construct. In contrast, the correlation between Factor 1 and Factor 2 was moderate (r = .51), supporting the presence of two related but empirically distinguishable dimensions.

Overall, these results provide further evidence for the multidimensional structure of the scale, while highlighting item 6 as a potential source of reduced measurement precision within Factor 2.

### 3.2 Item analysis for PS-8

Results are presented in **Table 1**. Descriptive statistics of the PS-8 items show that responses are adequately distributed and allow differentiation across varying levels of the construct being assessed, with no evidence of extreme ceiling or floor effects. The central tendency of the items corresponding to Factor 1 (items 1 to 4) falls within moderate values, whereas the items of Factor 2 (items 6 to 9) exhibit slightly higher means, suggesting that this dimension reflects a greater average intensity of the construct.

Regarding distribution, item skewness ranges from –0.974 to 0.856, with most items showing values close to zero, indicating approximately symmetrical distributions. Some items of Factor 2 display negative skewness, reflecting a slight concentration of responses at higher values, while certain items of Factor 1 show a slight concentration at lower values, without affecting the normality of the distributions. Item kurtosis ranges from –1.038 to 0.703, indicating relatively flat or slightly peaked distributions, all within acceptable limits.

Item psychometric analysis was conducted by examining item–rest correlations and Cronbach’s alpha if the item was deleted. Overall, item–rest correlations were adequate, ranging from.302 to.653, and largely exceeded the recommended minimum criterion of.30, indicating that the items contribute appropriately to the internal consistency of the scale. The items of F1 showed moderate to high item–rest correlations (.520–.653), demonstrating good discrimination and an adequate relationship with the overall construct. The items of F2 showed item–rest correlations ranging from moderate (items 7, 8, and 9:.452–.504) to low (item 6:.302), although still acceptable.

Removing item 6 resulted in only a modest increase in the alpha coefficient (α = .809), suggesting that although this item contributes less to the homogeneity of the scale, its negative impact is not sufficient to justify its removal from a psychometric perspective. Overall, the results support the psychometric adequacy of the items and justify their retention in the final version of the scale.

### 3.3 Reliability and internal validity of the PS-8

The results are presented in **Table 1**. The reliability of the PS was assessed using Cronbach’s alpha (α) and McDonald’s omega (ω) coefficients. Both the total PS score and the PS-F1 factor demonstrated good reliability (>. 70), indicating high internal consistency. In contrast, the PS-F2 factor showed lower, yet still acceptable, reliability values (>. 60). Internal validity (convergent and discriminant) was examined using the average variance extracted (AVE) and the HTMT index. The PS-F1 factor achieved an AVE value of.576, exceeding the recommended criterion of.50 and thus supporting adequate convergent validity. In addition, the HTMT value was well below the conservative threshold of.85, providing evidence of discriminant validity between the scale factors. By contrast, the PS-F2 factor showed an AVE value below the recommended criterion (AVE = .349), suggesting limited convergent validity. This finding is consistent with the lower reliability coefficients observed for this factor.

Overall, the results indicate that the PS exhibits adequate overall reliability and a satisfactory internal structure, with strong evidence of reliability and convergent and discriminant validity for the PS-F1 factor. Although acceptable from an exploratory perspective, the PS-F2 factor shows scope for psychometric improvement, which should be considered in future revisions of the scale.

### 3.4 External consistency of the PS-8

The results are presented in **Table 1**. The external consistency of the PS was examined using Pearson correlations with conceptually related scales (SABAS, BSMAS, GAS, and DLB). The use of Pearson’s correlation coefficient was justified by the fact that most of the analysed variables showed skewness and kurtosis values within acceptable ranges for parametric analyses. In addition, the scores corresponded to continuous variables measured on interval scales, and linear relationships between the constructs were assumed. Although the GAS scale showed skewness and kurtosis values that might suggest the use of non-parametric analyses, a parametric approach was retained for reasons of methodological consistency, as the remaining scales met the required assumptions. Moreover, Pearson’s correlation has been shown to be sufficiently robust to moderate deviations from normality, particularly in medium to large samples, and was therefore considered appropriate in this context.

The correlation results revealed a pattern of associations consistent with theoretical expectations. All correlations were statistically significant at the.01 level (two-tailed). The total PS score was positively and moderately correlated with SABAS and BSMAS, positively but weakly with GAS, and negatively, albeit weakly, with DLB. At the factorial level, PS-F1 and PS-F2 showed correlation patterns similar to that of the total scale, with slightly stronger associations for PS-F1. This pattern suggests that both factors contribute to the overall PS construct, albeit with different levels of intensity.

These results support both convergent and discriminant external validity, as SABAS, BSMAS, and GAS assess related constructs, whereas DLB measures a conceptually opposite construct. Overall, these findings provide solid evidence for the external consistency of the PS, supporting both its convergent and discriminant external validity and reinforcing its usefulness as a measure of the construct under study.

### 3.5 Sex invariance and construct for PS-8

The results of the factorial invariance analysis across sexes are presented in **Table 3**. The model showed an adequate fit at the configural level, indicating that the factorial structure is comparable for males and females. The progressive imposition of constraints did not lead to substantial deterioration in model fit, as changes in the fit indices (ΔCFI and ΔRMSEA) remained within recommended thresholds. Based on these criteria, evidence was found for metric, scalar, and strict invariance, supporting the equivalence of factor loadings, intercepts, and measurement errors across sexes. Thus, from a global model fit perspective, the instrument appears to assesses the construct equivalently in males and females, allowing for valid between-group comparisons.

**Table 3:** Sex invariance for PS-8 (Model 4) (n=396).

	Configural	Metric	Scalar	Strict
CFI	.941	.935	.930	.924
ΔCFI	--	-.006	-.005	-.006
RMSA	.093	.091	.088	.085
ΔRMSEA	--	-.002	-.003	-.003

CFI, Comparative Fit Index; ΔCFI, Delta Comparative Fit Index; RMSEA, Root Mean Square Error of Approximation; ΔRMSEA, Delta Root Mean Square Error of Approximation.

However, although the overall model fit is consistent with configural, metric, scalar, and strict invariance according to global fit indices, a more detailed inspection of the parameter estimates (available in the **Supplementary Materials**) suggests potential differences between groups.

However, although the overall model fit is consistent with configural metric, scalar, and strict invariance according to global fit indices, a more detailed inspection of the parameter estimates (available in the **Supplementary Materials**) suggests potential differences between groups. Specifically, in the female group (Group 2), the factor loadings of items 1 and 2 are significant and within expected ranges. In contrast, in the male group (Group 1), the loadings of the Factor 2 items are not significant (p >.05) and their confidence intervals are very wide, which may indicate that this factor is less clearly defined in this group.

The factor variance and covariance estimate, also indicate marked differences between groups, showing that F1 and F2 are related factors in the female group (F2 variance = .180, p = .004; covariance = .200, p <.001), whereas they are essentially independent factors in the male group (F2 variance = .032, p = .595; covariance = .007, p = .603).

Likewise, the correlation between factors differs across groups. In Group 2, the correlation between factors is moderate (.63), whereas in Group 1 it is very low (.08), indicating that F1 and F2 are virtually independent. These findings are in line with the HTMT results, which show adequate discriminant validity in Group 2 (0.645) but very low values in Group 1 (0.245), in line with the near-zero correlation between factors in this group. Taken together, these results reinforce the conclusion that the factorial structure is not equivalent across groups.

Taken together, these findings may indicate that the factorial structure is not fully equivalent across sexes. Therefore, despite the apparent support for strict invariance based on global fit indices, the overall pattern of results could be more compatible with partial measurement invariance, particularly with regard to Factor 2 in the male group. Accordingly, comparisons involving this factor should be interpreted with caution.

## 4 Discussion

The study’s objectives were largely achieved, although with some nuances that will be discussed in more detail below.

The present study aimed to identify the factorial model of the PS that best fits a Nigerian university student sample. Four models were evaluated: a unidimensional 10-item model, included for exploratory purposes; the original 10-item two-factor model validated by Karadağ et al. (1); the theoretical 10-item two-factor model with the original item assignment, supported in Spanish (20, 21) and Iranian samples (22); and the 8-item two-factor model (PS-8: F1: 1–4; F2: 6–9), proposed by Błachnio et al. (25) and replicated across multiple countries and age groups by Lin et al. (26)García-Castro et al. (27), and Kim et al. (28). Overall, the results suggest that the PS-8 provides the best fit for this population. In any case, the results of reliability and internal validity are heterogeneous. While Factor 1 (communication interference) showed good internal consistency and an adequate AVE value, Factor 2 (smartphone obsession) presented more modest reliability values and a low AVE, suggesting lower internal homogeneity. Meanwhile, the HTMT value and the correlation between factors support the presence of two related but empirically distinguishable dimensions, although the moderate values obtained for both parameters are noteworthy, indicating some divergence between factors. Similar findings have been reported in previous studies. Błachnio et al. (25) also reported moderate to low reliability indices for one or even both factors in some of the samples analysed in their study. Mendes et al. (23) described AVE values below.50. Furthermore, the observed correlation between factors is comparable to that reported by Kim et al. (28) and considerably higher than that reported by Zamani et al. (22). Overall, these results suggest that the PS-8 may be a reliable and valid instrument for assessing phubbing among Nigerian university students, particularly with respect to Factor 1, while Factor 2 shows room for psychometric improvement.

Regarding the second objective, strict invariance across sexes was formally supported in the present study based on global fit indices (ΔCFI and ΔRMSEA). These results are consistent with previous cross-cultural validations of the PS-8, which also reported scalar invariance across sexes. For example, Błachnio et al. (25) demonstrated that the 8-item two-factor structure exhibited scalar invariance in adult samples from 20 countries, while Lin et al. (26) replicated these findings in adults from Bangladesh, Iran, and Pakistan.

However, a more detailed inspection of the parameter estimates reveals substantial differences in the functioning of the second factor (smartphone obsession) between males and females. In females, the factor loadings of the F1 and F2 items were significant and within the expected ranges, with a moderate correlation between factors (r = 0.63) and an HTMT of 0.645, indicating related but empirically distinguishable dimensions. In contrast, in males, the loadings of the F2 items were not significant, showed very wide confidence intervals, the variance of Factor 2 was negligible, and the covariance between F1 and F2 was virtually zero, resulting in a near-zero correlation (r = .08) and an HTMT of.245. These findings suggest that, while F1 (communication interference) appears to function similarly across sexes, F2 may be weakly defined in males and shows limited association with F1. These results indicate that, despite the apparent support for strict invariance based on global fit indices, the phubbing construct, particularly the smartphone obsession dimension, may manifest differently between sexes.

This could be due to psychometric issues with the F2 items, as well as to potential differences in the construct structure between sexes. The low reliability (α and ω) and reduced AVE observed for F2 in both sexes suggest that the items of this factor may not adequately capture a coherent latent dimension. This could be due to unclear wording, items that may not fully capture the smartphone obsession construct, or items that may not reflect experiences that are equally relevant in this population. Items with limited clarity or cultural relevance may reduce a factor’s reliability and convergent validity. Overall, the observed low internal consistency and convergent validity suggest that F2 may not contribute in a stable way to the overall construct, which limits the interpretability of this dimension.

Beyond item-related issues, the pattern observed in male group suggest that F2 may not operates in the same way as in females. In the male group, F2 to be weakly related to F1, with a near-zero correlation and very low HTMT values, whereas in female, both factors show a moderate correlation and an HTMT consistent with related but empirically distinguishable dimensions. According to Brown & Moore (43) and Indu et al. (44), this may reflect differences in the interpretation, relevance, or integration of the items within the construct; which, as suggested by Wood and Eagly (45), can be consistent with evidence that psychological constructs can manifest differently across subgroups, such as sex or culture.

Overall, these findings suggest that, although global results allow for the assumption of strict invariance, the F2 dimension should be interpreted with caution. It may be necessary to consider the revision or adaptation of items to improve reliability, or alternatively, the possibility that the structure of the phubbing construct may present conceptual differences between males and females, particularly in how smartphone obsession relates to communication interference. Taken together, the present findings suggest that Smartphone Obsession should not be interpreted merely as a psychometrically weak or context-specific dimension, but rather as a potentially more fragile component of the Phubbing Scale across cultures and sexes. The observed instability in the Nigerian sample appears to be consistent with findings from other validation studies, suggesting a broader cross-cultural pattern rather than an isolated anomaly (e.g., 22, 23, 28).

In this sense, the Nigerian data may be particularly illustrative of a general theoretical issue: Smartphone Obsession may reflect a more fluid and culturally contingent manifestation of phubbing, which is potentially sensitive to social norms, technological practices, and contextual meanings attached to smartphone use.

This heterogeneity in results, observed in the present study and across previous validation studies in the literature, may reflect cultural influences on the perception and experience of phubbing, particularly smartphone obsession. It should be noted that, according Hofstede (46), although phubbing appears to be a transcultural phenomenon, in collectivist cultures such as Nigeria, simultaneous phone use may be perceived as disruptive, which, as suggested by Büttner et al. (47), may explain why individuals in these contexts tend to report a higher perception of phubbing than those in more individualistic countries. According to Garris et al. (48)Uskul and Over (49), and others, this response may be due to the fact that individuals from collectivist environments generally exhibit greater sensitivity to rejection. This, in turn, according to Zimmer-Gembeck et al. (50), may lead them to attribute experiences of exclusion, such as phubbing, to personal shortcomings rather than external circumstances. Thus, this cultural sensitivity may help to explain why behaviours associated with communication interference (Factor 1) exhibit more robust and consistent psychometric properties in this sample. In contrast, the weaker and less stable performance of the smartphone obsession dimension (Factor 2) may indicate that the internal or motivational aspects of phubbing are less clearly defined or less salient in this cultural context. Taken together, these findings suggest that, in this context, phubbing may be understood primarily as a relational phenomenon (i.e. centred on its social consequences), rather than as a behaviour driven by an internal dependence on the smartphone. This interpretation is consistent with the stronger performance of Factor 1 relative to Factor 2 observed in the present study.

Regarding sex differences, the findings of the present study further suggest that the smartphone obsession dimension does not operate equivalently in male and female. This is consistent with previous research, such as Randler et al. (51), which identifies sex as a significant predictor of problematic or addictive technology use, as well as with the conceptualisation proposed by Karadağ et al. (1), according to which phubbing not only involves observable social interference but is also driven by underlying motivational factors related to smartphone use. In this regard, Lanette et al. (52) report key findings from interview-based research exploring smartphone obsession. A secondary analysis of their results suggests potential differences in how males and females conceptualise smartphone-related behaviours.

Female may tend to internalise dominant social narratives surrounding technological addiction, potentially linking smartphone use to feelings of guilt, responsibility, and emotional strain, particularly in relation to family and social interaction. For them, smartphone obsession may not merely be perceived as a habit, but as a factor that could affect their well-being and relationships, generating ongoing concern about how their behaviour is evaluated by others. By contrast, male may be more likely to evaluate the same behaviour from a functional or pragmatic perspective, interpreting intensity of use primarily in terms of control, convenience, or efficiency, and are less prone to experience guilt or internal distress associated with smartphone use. From a theoretical perspective, these findings may be consistent with the idea that phubbing is not a uniform construct, but rather one whose internal structure may vary across subgroups. In particular, the results suggest that the motivational dimension of phubbing (smartphone obsession) may be more salient and more psychologically integrated in female than in male.

## 5 Practical implications

From both an applied and a theoretical perspective, these results support the use of the PS-8 for assessing phubbing among Nigerian university students, particularly through the communication interference factor, which showed more robust and consistent functioning. However, the findings also highlight the need to interpret the smartphone obsession dimension with caution, given its weaker psychometric performance and its differential functioning across sexes.

In this regard, the results suggest that future research should further examine the conceptual and psychometric adequacy of this dimension, either by revising or reformulating its items, or by exploring whether smartphone obsession is integrated differently into the phubbing construct depending on sex or cultural context. Such work would contribute to more precise and culturally sensitive measurement and intervention approaches across diverse populations.

In terms of intervention design, the prominence of this factor suggests that strategies should primarily focus on the interpersonal consequences of smartphone use during face-to-face interactions. Accordingly, educational and digital well-being programmes may benefit from emphasising norms of attentiveness, respect, and presence in social contexts. Data also can suggests that interventions focused exclusively on smartphone dependence may be less effective in this population. Instead, approaches targeting behavioural regulation in social contexts, such as promoting phone-free interaction periods or mindful smartphone use, may be more appropriate. In addition, the observed sex differences indicate that intervention strategies may need to be tailored. Emotional and relational components may be more relevant for women, whereas behavioural and self-regulation strategies may be more suitable for men.

## 6 Strengths and limitations

The present study has several strengths. First, it contributes to the cross-cultural validation of the PS-8 by providing evidence from a Nigerian university student sample, an under-represented context in the phubbing literature. Second, it applies a rigorous psychometric approach, including the comparison of multiple factorial models, which strengthens the robustness of the findings. Third, the inclusion of measurement invariance testing across sex allows for a more precise evaluation of the equivalence of the construct across groups. Finally, the study contributes both theoretically and practically by highlighting potential cultural and sex-related differences in the structure of phubbing and informing future assessment and intervention strategies.

Despite these strengths, several limitations should be acknowledged when interpreting the findings. First, the use of a non-probability convenience sampling method limits the representativeness of the sample and, consequently, the generalisability of the results beyond the specific group of Nigerian university students included in this study. In this regard, the findings should be interpreted within the Nigerian cultural and educational context, and caution is warranted when extrapolating them to other populations, age groups, or cultural settings.

Second, the marked imbalance in the sex distribution of the sample, with a substantially higher proportion of female participants, represents an important limitation, particularly for the multigroup analyses. Although strict measurement invariance across sex was supported by global fit indices, the reduced size of the male subsample may have affected the stability and precision of parameter estimates in this group.

Finally, all measures relied exclusively on self-report instruments, which may be subject to biases such as social desirability, recall errors, or subjective interpretation of the items, especially when assessing behaviours related to smartphone use.

## 7 Conclusion

Despite these limitations, the present study provides important evidence regarding the psychometric properties of the Phubbing Scale (PS) in a Nigerian university student sample. The results indicate that the 8-item two-factor version (PS-8) offers the best fit, supporting its use as a potentially useful instrument for assessing phubbing in this population. Factor 1 (communication interference) demonstrated good internal consistency and convergent validity, whereas Factor 2 (smartphone obsession) showed lower reliability and AVE values, suggesting that caution is needed when interpreting this dimension.

Strict measurement invariance across sex was generally supported based on global fit indices, allowing for comparisons between male and female students at a global level. However, inspection of parameter estimates revealed that Factor 2 is poorly defined in males. with weak or non-significant loadings, suggesting potential differences in the functioning of this dimension across sexes. These findings indicate that, although the PS-8 may be useful for research and interventions in Nigerian universities, future studies should consider refining the items of Factor 2 and further exploring possible sex-related and cultural variations in the phubbing construct.

Overall, this study contributes to the cross-cultural validation of the PS-8, expanding its applicability and providing a basis for further research on phubbing and its psychosocial correlates in Nigerian and other African contexts.

## Data Availability

The raw data supporting the conclusions of this article will be made available by the authors, without undue reservation.
