# Supplementary material for: Does the phubbing scale measure the same construct across sexes? Evidence from a Nigerian sample
Source: Front Psychiatry. 2026 May 22;17:1820789. doi: 10.3389/fpsyt.2026.1820789 (PMC13236879; doi:10.3389/fpsyt.2026.1820789)

**Does the Phubbing Scale (PS) Measure the Same Construct Across Sexes? Evidence From a Nigerian Sample**

**SUPPLEMENTARY MATERIALS**

## PS-8 (F1: items 1,2,3,4; F2: items 6,7,8,9)

## Confirmatory Factor Analysis

| *Chi-square test* | | | | | | | |
| --- | --- | --- | --- | --- | --- | --- | --- |
| Model | | Χ² | | df | | p | |
| Baseline model |  | 1,090.282 |  | 28 |  |  |  |
| Factor model |  | 73.213 |  | 19 |  | < .001 |  |
|  | | | | | | | |
| Note.   The estimator is ML. The test statistic is standard. The standard error method is standard. | | | | | | | |

| *Fit indices* | | | | | | |
| --- | --- | --- | --- | --- | --- | --- |
| Index | | Value | | | | |
| Comparative Fit Index (CFI) |  | 0.949 | | |  | |
| Tucker-Lewis Index (TLI) |  | 0.925 | | |  | |
|  | | | | | | |
| Root mean square error of approximation (RMSEA) | | |  | 0.085 | |  |
| RMSEA 90% CI lower bound | | |  | 0.065 | |  |
| RMSEA 90% CI upper bound | | |  | 0.106 | |  |
| RMSEA p-value | | |  | 0.003 | |  |
| Standardized root mean square residual (SRMR) | | |  | 0.051 | |  |
|  | | | | | | |

| *Factor loadings* | | | | | | | | | | | | | | | |
| --- | --- | --- | --- | --- | --- | --- | --- | --- | --- | --- | --- | --- | --- | --- | --- |
|  | | | | | | | | | | | | 95% Confidence Interval | | | |
| Factor | | Indicator | | Estimate | | Std. Error | | z-value | | p | | Lower | | Upper | |
| Factor 1 |  | PS-1 |  | 1.000 |  | 0.000 |  |  |  |  |  | 1.000 |  | 1.000 |  |
|  |  | PS-2 |  | 1.183 |  | 0.089 |  | 13.259 |  | < .001 |  | 1.008 |  | 1.358 |  |
|  |  | PS-3 |  | 0.940 |  | 0.083 |  | 11.276 |  | < .001 |  | 0.776 |  | 1.103 |  |
|  |  | PS-4 |  | 1.205 |  | 0.090 |  | 13.336 |  | < .001 |  | 1.028 |  | 1.382 |  |
| Factor 2 |  | PS-6 |  | 1.000 |  | 0.000 |  |  |  |  |  | 1.000 |  | 1.000 |  |
|  |  | PS-7 |  | 1.489 |  | 0.270 |  | 5.507 |  | < .001 |  | 0.959 |  | 2.018 |  |
|  |  | PS-8 |  | 1.818 |  | 0.329 |  | 5.527 |  | < .001 |  | 1.173 |  | 2.463 |  |
|  |  | PS-9 |  | 1.990 |  | 0.354 |  | 5.614 |  | < .001 |  | 1.295 |  | 2.685 |  |
|  | | | | | | | | | | | | | | | |

| *Factor variances* | | | | | | | | | | | | | |
| --- | --- | --- | --- | --- | --- | --- | --- | --- | --- | --- | --- | --- | --- |
|  | | | | | | | | | | 95% Confidence Interval | | | |
| Factor | | Estimate | | Std. Error | | z-value | | p | | Lower | | Upper | |
| Factor 1 |  | 0.494 |  | 0.075 |  | 6.624 |  | < .001 |  | 0.348 |  | 0.640 |  |
| Factor 2 |  | 0.171 |  | 0.057 |  | 2.995 |  | .003 |  | 0.059 |  | 0.283 |  |
|  | | | | | | | | | | | | | |

| *Factor Covariances* | | | | | | | | | | | | | | | | | | | | | | | |  |
| --- | --- | --- | --- | --- | --- | --- | --- | --- | --- | --- | --- | --- | --- | --- | --- | --- | --- | --- | --- | --- | --- | --- | --- | --- |
|  | | | | | | | | | | | | | | | | | | | 95% Confidence Interval | | | | |  |
|  | |  | | |  | | Estimate | | | | Std. Error | | | z-value | | p | | | Lower | | | Upper | |  |
| Factor 1 |  | | ↔ |  | | Factor 2 | |  | 0.152 |  | | 0.033 |  | | 4.617 | |  | < .001 | |  | 0.087 |  | 0.216 |  |
|  | | | | | | | | | | | | | | | | | | | | | | | |  |

| *Residual variances* | | | | | | | | | | | | | |
| --- | --- | --- | --- | --- | --- | --- | --- | --- | --- | --- | --- | --- | --- |
|  | | | | | | | | | | 95% Confidence Interval | | | |
| Indicator | | Estimate | | Std. Error | | z-value | | p | | Lower | | Upper | |
| PS-1 |  | 0.755 |  | 0.059 |  | 12.870 |  | < .001 |  | 0.640 |  | 0.870 |  |
| PS-2 |  | 0.241 |  | 0.029 |  | 8.168 |  | < .001 |  | 0.183 |  | 0.298 |  |
| PS-3 |  | 0.508 |  | 0.041 |  | 12.480 |  | < .001 |  | 0.428 |  | 0.587 |  |
| PS-4 |  | 0.221 |  | 0.029 |  | 7.551 |  | < .001 |  | 0.164 |  | 0.278 |  |
| PS-6 |  | 1.305 |  | 0.097 |  | 13.415 |  | < .001 |  | 1.114 |  | 1.495 |  |
| PS-7 |  | 0.570 |  | 0.054 |  | 10.585 |  | < .001 |  | 0.464 |  | 0.676 |  |
| PS-8 |  | 0.804 |  | 0.077 |  | 10.370 |  | < .001 |  | 0.652 |  | 0.955 |  |
| PS-9 |  | 0.663 |  | 0.076 |  | 8.721 |  | < .001 |  | 0.514 |  | 0.811 |  |
|  | | | | | | | | | | | | | |

#### Model plot


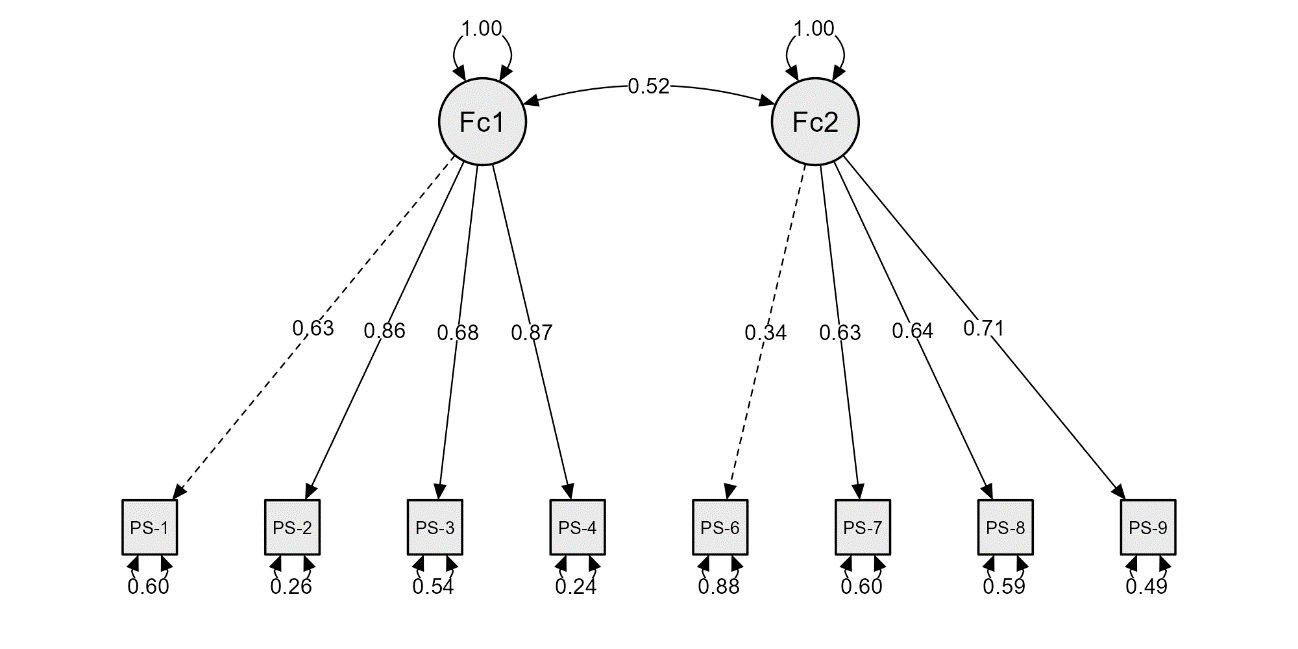


## PS-8 (F1: items 1,2,3,4; F2: items 6,7,8,9)

## Configural Invariance

| *Chi-square test* | | | | | | | |
| --- | --- | --- | --- | --- | --- | --- | --- |
| Model | | Χ² | | df | | p | |
| Baseline model |  | 1,158.025 |  | 56 |  |  |  |
| Factor model |  | 102.808 |  | 38 |  | < .001 |  |
|  | | | | | | | |
| Note.   The estimator is ML. The test statistic is standard. The standard error method is standard. | | | | | | | |

####

| *Fit indices* | | | | | | |
| --- | --- | --- | --- | --- | --- | --- |
| Index | | | Value | | | |
| Comparative Fit Index (CFI) |  | | 0.941 | |  | |
| Tucker-Lewis Index (TLI) |  | | 0.913 | |  | |
|  | | | | | | |
| Root mean square error of approximation (RMSEA) | |  | | 0.093 | |  |
| RMSEA 90% CI lower bound | |  | | 0.071 | |  |
| RMSEA 90% CI upper bound | |  | | 0.115 | |  |
| RMSEA p-value | |  | | 8.427×10^-4^ | |  |
| Standardized root mean square residual (SRMR) | |  | | 0.061 | |  |
|  | | | | | | |

| *Factor loadings* | | | | | | | | | | | | | | | | | |
| --- | --- | --- | --- | --- | --- | --- | --- | --- | --- | --- | --- | --- | --- | --- | --- | --- | --- |
|  | | | | | | | | | | | | | | 95% Confidence Interval | | | |
| Group | | Factor | | Indicator | | Estimate | | Std. Error | | z-value | | p | | Lower | | Upper | |
| 2 |  | Factor 1 |  | PS-1 |  | 1.000 |  | 0.000 |  |  |  |  |  | 1.000 |  | 1.000 |  |
|  |  |  |  | PS-2 |  | 1.068 |  | 0.084 |  | 12.748 |  | < .001 |  | 0.904 |  | 1.233 |  |
|  |  |  |  | PS-3 |  | 0.915 |  | 0.082 |  | 11.183 |  | < .001 |  | 0.754 |  | 1.075 |  |
|  |  |  |  | PS-4 |  | 1.109 |  | 0.085 |  | 12.999 |  | < .001 |  | 0.942 |  | 1.277 |  |
|  |  | Factor 2 |  | PS-6 |  | 1.000 |  | 0.000 |  |  |  |  |  | 1.000 |  | 1.000 |  |
|  |  |  |  | PS-7 |  | 1.414 |  | 0.271 |  | 5.219 |  | < .001 |  | 0.883 |  | 1.944 |  |
|  |  |  |  | PS-8 |  | 1.750 |  | 0.333 |  | 5.262 |  | < .001 |  | 1.098 |  | 2.402 |  |
|  |  |  |  | PS-9 |  | 1.898 |  | 0.355 |  | 5.354 |  | < .001 |  | 1.203 |  | 2.593 |  |
| 1 |  | Factor 1 |  | PS-1 |  | 1.000 |  | 0.000 |  |  |  |  |  | 1.000 |  | 1.000 |  |
|  |  |  |  | PS-2 |  | 1.898 |  | 0.433 |  | 4.388 |  | < .001 |  | 1.050 |  | 2.746 |  |
|  |  |  |  | PS-3 |  | 1.110 |  | 0.304 |  | 3.647 |  | < .001 |  | 0.513 |  | 1.706 |  |
|  |  |  |  | PS-4 |  | 1.667 |  | 0.378 |  | 4.414 |  | < .001 |  | 0.927 |  | 2.408 |  |
|  |  | Factor 2 |  | PS-6 |  | 1.000 |  | 0.000 |  |  |  |  |  | 1.000 |  | 1.000 |  |
|  |  |  |  | PS-7 |  | 3.081 |  | 2.938 |  | 1.048 |  | .294 |  | -2.678 |  | 8.839 |  |
|  |  |  |  | PS-8 |  | 4.729 |  | 4.469 |  | 1.058 |  | .290 |  | -4.029 |  | 13.488 |  |
|  |  |  |  | PS-9 |  | 5.665 |  | 5.375 |  | 1.054 |  | .292 |  | -4.870 |  | 16.200 |  |
|  | | | | | | | | | | | | | | | | | |

| *Factor variances* | | | | | | | | | | | | | | | |
| --- | --- | --- | --- | --- | --- | --- | --- | --- | --- | --- | --- | --- | --- | --- | --- |
|  | | | | | | | | | | | | 95% Confidence Interval | | | |
| Group | | Factor | | Estimate | | Std. Error | | z-value | | p | | Lower | | Upper | |
| 2 |  | Factor 1 |  | 0.560 |  | 0.087 |  | 6.438 |  | < .001 |  | 0.390 |  | 0.731 |  |
|  |  | Factor 2 |  | 0.180 |  | 0.063 |  | 2.872 |  | .004 |  | 0.057 |  | 0.303 |  |
| 1 |  | Factor 1 |  | 0.269 |  | 0.123 |  | 2.189 |  | .029 |  | 0.028 |  | 0.510 |  |
|  |  | Factor 2 |  | 0.032 |  | 0.060 |  | 0.532 |  | .595 |  | -0.086 |  | 0.150 |  |
|  | | | | | | | | | | | | | | | |

| *Factor Covariances* | | | | | | | | | | | | | | | | | | | | |  |
| --- | --- | --- | --- | --- | --- | --- | --- | --- | --- | --- | --- | --- | --- | --- | --- | --- | --- | --- | --- | --- | --- |
|  | | | | | | | | | | | | | | | | 95% Confidence Interval | | | | |  |
| Group | |  | |  | |  | | Estimate | | Std. Error | | z-value | | p | | | Lower | | Upper | | |
| 2 |  | Factor 1 |  | ↔ |  | Factor 2 |  | 0.200 |  | 0.043 |  | 4.598 |  | < .001 |  | | 0.115 |  | 0.285 |  | |
| 1 |  | Factor 1 |  | ↔ |  | Factor 2 |  | 0.007 |  | 0.014 |  | 0.520 |  | .603 |  | | -0.020 |  | 0.035 |  | |
|  | | | | | | | | | | | | | | | | | | | | |  |

| *Residual variances* | | | | | | | | | | | | | | | |
| --- | --- | --- | --- | --- | --- | --- | --- | --- | --- | --- | --- | --- | --- | --- | --- |
|  | | | | | | | | | | | | 95% Confidence Interval | | | |
| Group | | Indicator | | Estimate | | Std. Error | | z-value | | p | | Lower | | Upper | |
| 2 |  | PS-1 |  | 0.692 |  | 0.061 |  | 11.301 |  | < .001 |  | 0.572 |  | 0.812 |  |
|  |  | PS-2 |  | 0.259 |  | 0.031 |  | 8.265 |  | < .001 |  | 0.198 |  | 0.320 |  |
|  |  | PS-3 |  | 0.449 |  | 0.041 |  | 10.899 |  | < .001 |  | 0.368 |  | 0.529 |  |
|  |  | PS-4 |  | 0.214 |  | 0.030 |  | 7.091 |  | < .001 |  | 0.155 |  | 0.273 |  |
|  |  | PS-6 |  | 1.209 |  | 0.101 |  | 11.993 |  | < .001 |  | 1.012 |  | 1.407 |  |
|  |  | PS-7 |  | 0.572 |  | 0.058 |  | 9.851 |  | < .001 |  | 0.458 |  | 0.685 |  |
|  |  | PS-8 |  | 0.787 |  | 0.083 |  | 9.522 |  | < .001 |  | 0.625 |  | 0.949 |  |
|  |  | PS-9 |  | 0.672 |  | 0.080 |  | 8.354 |  | < .001 |  | 0.514 |  | 0.830 |  |
| 1 |  | PS-1 |  | 0.864 |  | 0.143 |  | 6.028 |  | < .001 |  | 0.583 |  | 1.145 |  |
|  |  | PS-2 |  | 0.092 |  | 0.093 |  | 0.985 |  | .325 |  | -0.091 |  | 0.274 |  |
|  |  | PS-3 |  | 0.716 |  | 0.121 |  | 5.916 |  | < .001 |  | 0.479 |  | 0.954 |  |
|  |  | PS-4 |  | 0.326 |  | 0.089 |  | 3.677 |  | < .001 |  | 0.152 |  | 0.499 |  |
|  |  | PS-6 |  | 1.801 |  | 0.291 |  | 6.182 |  | < .001 |  | 1.230 |  | 2.372 |  |
|  |  | PS-7 |  | 0.679 |  | 0.126 |  | 5.392 |  | < .001 |  | 0.432 |  | 0.926 |  |
|  |  | PS-8 |  | 0.779 |  | 0.192 |  | 4.054 |  | < .001 |  | 0.402 |  | 1.155 |  |
|  |  | PS-9 |  | 0.283 |  | 0.213 |  | 1.328 |  | .184 |  | -0.134 |  | 0.700 |  |
|  | | | | | | | | | | | | | | | |

| *Average variance extracted* | | | | | | | | | | | | | | | | | | | |
| --- | --- | --- | --- | --- | --- | --- | --- | --- | --- | --- | --- | --- | --- | --- | --- | --- | --- | --- | --- |
| Group | | | | | | Factor | | | | | | | | AVE | | | | | |
| 2 | | | |  | | Factor 1 | | | | |  | | | 0.594 | | |  | | |
|  | | | |  | | Factor 2 | | | | |  | | | 0.349 | | |  | | |
| 1 | | | |  | | Factor 1 | | | | |  | | | 0.537 | | |  | | |
|  | | | |  | | Factor 2 | | | | |  | | | 0.371 | | |  | | |
|  | | | | | | | | | | | | | | | | | | | |
| *Heterotrait-monotrait ratio* | | | | | | | | | | | | | | | | | | | |
| Group | | | | |  | | | | | Factor 1 | | | | | | Factor 2 | | | |
| 2 | |  | | | Factor 1 | | | |  | 1.000 | | |  | | |  | | |  |
|  | |  | | | Factor 2 | | | |  | 0.645 | | |  | | | 1.000 | | |  |
| 1 | |  | | | Factor 1 | | | |  | 1.000 | | |  | | |  | | |  |
|  | |  | | | Factor 2 | | | |  | 0.245 | | |  | | | 1.000 | | |  |
|  | | | | | | | | | | | | | | | | | | | |
| *Reliability* | | | | | | | | | | | | | | | | | | | |
| Group | | |  | | | | | Coefficient ω | | | | | | | Coefficient α | | | | |
| 2 |  | | Factor 1 | | | |  | 0.855 | | | |  | | | 0.851 | | |  | |
|  |  | | Factor 2 | | | |  | 0.670 | | | |  | | | 0.661 | | |  | |
|  |  | | total | | | |  | 0.829 | | | |  | | | 0.816 | | |  | |
| 1 |  | | Factor 1 | | | |  | 0.828 | | | |  | | | 0.783 | | |  | |
|  |  | | Factor 2 | | | |  | 0.640 | | | |  | | | 0.619 | | |  | |
|  |  | | total | | | |  | 0.624 | | | |  | | | 0.715 | | |  | |
|  | | | | | | | | | | | | | | | | | | | |

####

#### Model plots

##### 2


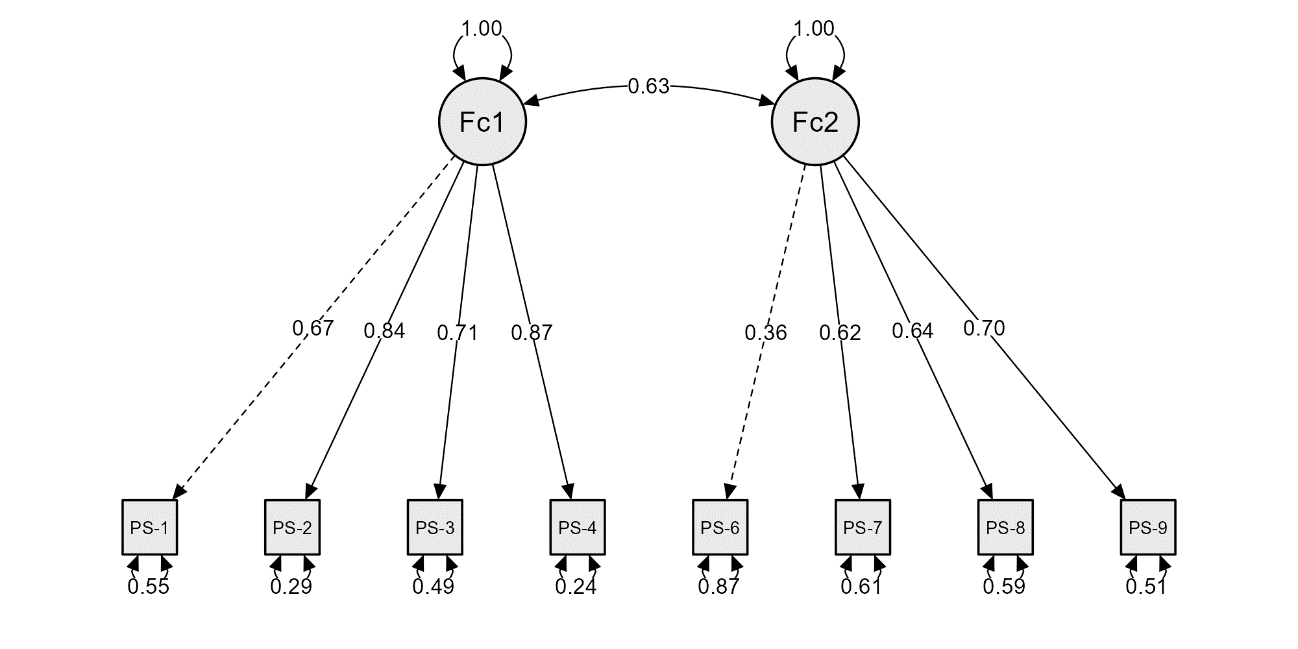


##### 1


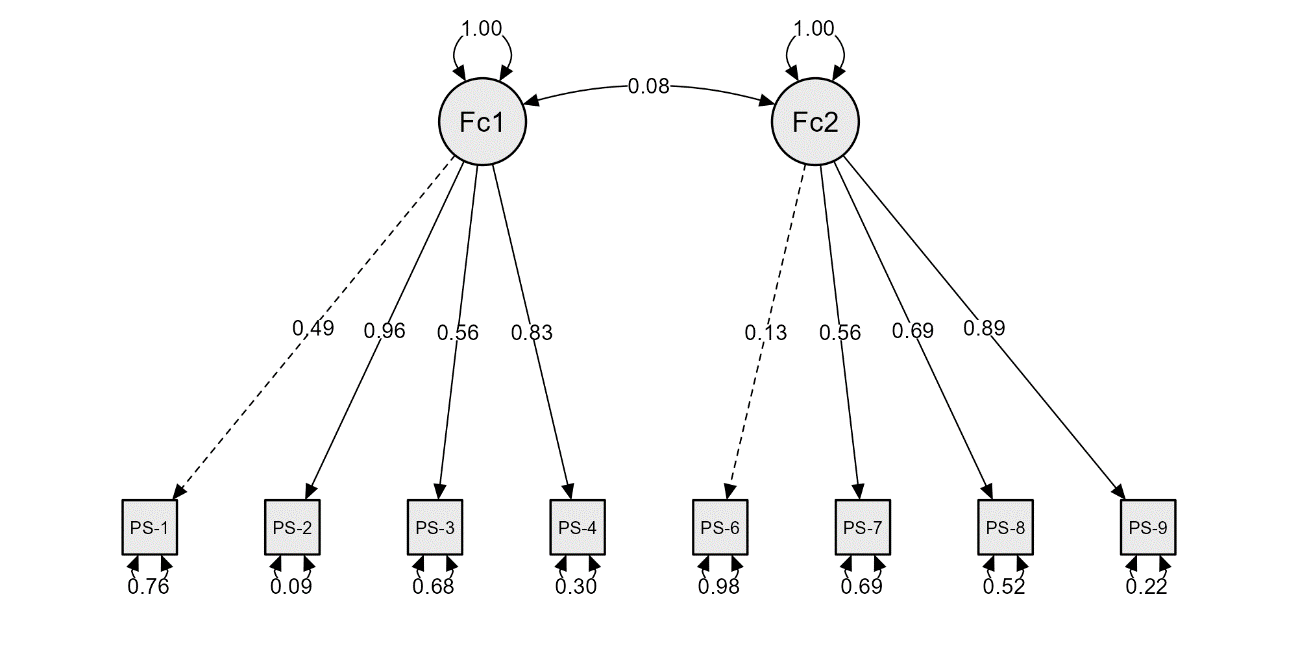


## PS-8 (F1: items 1,2,3,4; F2: items 6,7,8,9)

## Metric Invariance

| *Chi-square test* | | | | | | | |
| --- | --- | --- | --- | --- | --- | --- | --- |
| Model | | Χ² | | df | | p | |
| Baseline model |  | 1,158.025 |  | 56 |  |  |  |
| Factor model |  | 115.784 |  | 44 |  | < .001 |  |
|  | | | | | | | |
| Note.   The estimator is ML. The test statistic is standard. The standard error method is standard. | | | | | | | |

| *Fit indices* | | | | | | |
| --- | --- | --- | --- | --- | --- | --- |
| Index | | | Value | | | |
| Comparative Fit Index (CFI) |  | | 0.935 | |  | |
| Tucker-Lewis Index (TLI) |  | | 0.917 | |  | |
|  | | | | | | |
| Root mean square error of approximation (RMSEA) | |  | | 0.091 | |  |
| RMSEA 90% CI lower bound | |  | | 0.071 | |  |
| RMSEA 90% CI upper bound | |  | | 0.111 | |  |
| RMSEA p-value | |  | | 6.921×10^-4^ | |  |
| Standardized root mean square residual (SRMR) | |  | | 0.071 | |  |
|  | | | | | | |

| *Factor loadings* | | | | | | | | | | | | | | | | | |
| --- | --- | --- | --- | --- | --- | --- | --- | --- | --- | --- | --- | --- | --- | --- | --- | --- | --- |
|  | | | | | | | | | | | | | | 95% Confidence Interval | | | |
| Group | | Factor | | Indicator | | Estimate | | Std. Error | | z-value | | p | | Lower | | Upper | |
| 2 |  | Factor 1 |  | PS-1 |  | 1.000 |  | 0.000 |  |  |  |  |  | 1.000 |  | 1.000 |  |
|  |  |  |  | PS-2 |  | 1.177 |  | 0.087 |  | 13.469 |  | < .001 |  | 1.006 |  | 1.348 |  |
|  |  |  |  | PS-3 |  | 0.947 |  | 0.082 |  | 11.553 |  | < .001 |  | 0.786 |  | 1.108 |  |
|  |  |  |  | PS-4 |  | 1.194 |  | 0.088 |  | 13.579 |  | < .001 |  | 1.022 |  | 1.366 |  |
|  |  | Factor 2 |  | PS-6 |  | 1.000 |  | 0.000 |  |  |  |  |  | 1.000 |  | 1.000 |  |
|  |  |  |  | PS-7 |  | 1.524 |  | 0.290 |  | 5.249 |  | < .001 |  | 0.955 |  | 2.093 |  |
|  |  |  |  | PS-8 |  | 1.987 |  | 0.373 |  | 5.333 |  | < .001 |  | 1.257 |  | 2.717 |  |
|  |  |  |  | PS-9 |  | 2.174 |  | 0.403 |  | 5.398 |  | < .001 |  | 1.385 |  | 2.964 |  |
| 1 |  | Factor 1 |  | PS-1 |  | 1.000 |  | 0.000 |  |  |  |  |  | 1.000 |  | 1.000 |  |
|  |  |  |  | PS-2 |  | 1.177 |  | 0.087 |  | 13.469 |  | < .001 |  | 1.006 |  | 1.348 |  |
|  |  |  |  | PS-3 |  | 0.947 |  | 0.082 |  | 11.553 |  | < .001 |  | 0.786 |  | 1.108 |  |
|  |  |  |  | PS-4 |  | 1.194 |  | 0.088 |  | 13.579 |  | < .001 |  | 1.022 |  | 1.366 |  |
|  |  | Factor 2 |  | PS-6 |  | 1.000 |  | 0.000 |  |  |  |  |  | 1.000 |  | 1.000 |  |
|  |  |  |  | PS-7 |  | 1.524 |  | 0.290 |  | 5.249 |  | < .001 |  | 0.955 |  | 2.093 |  |
|  |  |  |  | PS-8 |  | 1.987 |  | 0.373 |  | 5.333 |  | < .001 |  | 1.257 |  | 2.717 |  |
|  |  |  |  | PS-9 |  | 2.174 |  | 0.403 |  | 5.398 |  | < .001 |  | 1.385 |  | 2.964 |  |
|  | | | | | | | | | | | | | | | | | |

| *Factor variances* | | | | | | | | | | | | | | | |
| --- | --- | --- | --- | --- | --- | --- | --- | --- | --- | --- | --- | --- | --- | --- | --- |
|  | | | | | | | | | | | | 95% Confidence Interval | | | |
| Group | | Factor | | Estimate | | Std. Error | | z-value | | p | | Lower | | Upper | |
| 2 |  | Factor 1 |  | 0.488 |  | 0.075 |  | 6.510 |  | < .001 |  | 0.341 |  | 0.635 |  |
|  |  | Factor 2 |  | 0.144 |  | 0.051 |  | 2.819 |  | .005 |  | 0.044 |  | 0.245 |  |
| 1 |  | Factor 1 |  | 0.554 |  | 0.123 |  | 4.516 |  | < .001 |  | 0.313 |  | 0.794 |  |
|  |  | Factor 2 |  | 0.177 |  | 0.071 |  | 2.483 |  | .013 |  | 0.037 |  | 0.317 |  |
|  | | | | | | | | | | | | | | | |

| *Factor Covariances* | | | | | | | | | | | | | | | | | | | |
| --- | --- | --- | --- | --- | --- | --- | --- | --- | --- | --- | --- | --- | --- | --- | --- | --- | --- | --- | --- |
|  | | | | | | | | | | | | | | | | 95% Confidence Interval | | | |
| Group | |  | |  | |  | | Estimate | | Std. Error | | z-value | | p | | Lower | | Upper | |
| 2 |  | Factor 1 |  | ↔ |  | Factor 2 |  | 0.166 |  | 0.037 |  | 4.549 |  | < .001 |  | 0.095 |  | 0.238 |  |
| 1 |  | Factor 1 |  | ↔ |  | Factor 2 |  | 0.040 |  | 0.044 |  | 0.922 |  | .357 |  | -0.045 |  | 0.126 |  |
|  | | | | | | | | | | | | | | | | | | | |

| *Residual variances* | | | | | | | | | | | | | | | |
| --- | --- | --- | --- | --- | --- | --- | --- | --- | --- | --- | --- | --- | --- | --- | --- |
|  | | | | | | | | | | | | 95% Confidence Interval | | | |
| Group | | Indicator | | Estimate | | Std. Error | | z-value | | p | | Lower | | Upper | |
| 2 |  | PS-1 |  | 0.705 |  | 0.061 |  | 11.533 |  | < .001 |  | 0.585 |  | 0.824 |  |
|  |  | PS-2 |  | 0.252 |  | 0.032 |  | 7.978 |  | < .001 |  | 0.190 |  | 0.313 |  |
|  |  | PS-3 |  | 0.456 |  | 0.041 |  | 11.087 |  | < .001 |  | 0.375 |  | 0.536 |  |
|  |  | PS-4 |  | 0.212 |  | 0.030 |  | 7.073 |  | < .001 |  | 0.153 |  | 0.270 |  |
|  |  | PS-6 |  | 1.223 |  | 0.101 |  | 12.150 |  | < .001 |  | 1.026 |  | 1.421 |  |
|  |  | PS-7 |  | 0.584 |  | 0.057 |  | 10.243 |  | < .001 |  | 0.472 |  | 0.696 |  |
|  |  | PS-8 |  | 0.778 |  | 0.082 |  | 9.544 |  | < .001 |  | 0.618 |  | 0.938 |  |
|  |  | PS-9 |  | 0.655 |  | 0.080 |  | 8.218 |  | < .001 |  | 0.499 |  | 0.811 |  |
| 1 |  | PS-1 |  | 0.889 |  | 0.154 |  | 5.766 |  | < .001 |  | 0.587 |  | 1.191 |  |
|  |  | PS-2 |  | 0.197 |  | 0.061 |  | 3.213 |  | .001 |  | 0.077 |  | 0.318 |  |
|  |  | PS-3 |  | 0.704 |  | 0.123 |  | 5.707 |  | < .001 |  | 0.462 |  | 0.946 |  |
|  |  | PS-4 |  | 0.264 |  | 0.069 |  | 3.805 |  | < .001 |  | 0.128 |  | 0.400 |  |
|  |  | PS-6 |  | 1.786 |  | 0.295 |  | 6.064 |  | < .001 |  | 1.209 |  | 2.364 |  |
|  |  | PS-7 |  | 0.628 |  | 0.121 |  | 5.198 |  | < .001 |  | 0.391 |  | 0.864 |  |
|  |  | PS-8 |  | 0.758 |  | 0.160 |  | 4.725 |  | < .001 |  | 0.444 |  | 1.073 |  |
|  |  | PS-9 |  | 0.431 |  | 0.135 |  | 3.199 |  | .001 |  | 0.167 |  | 0.696 |  |
|  | | | | | | | | | | | | | | | |

#### Model plots

##### 2


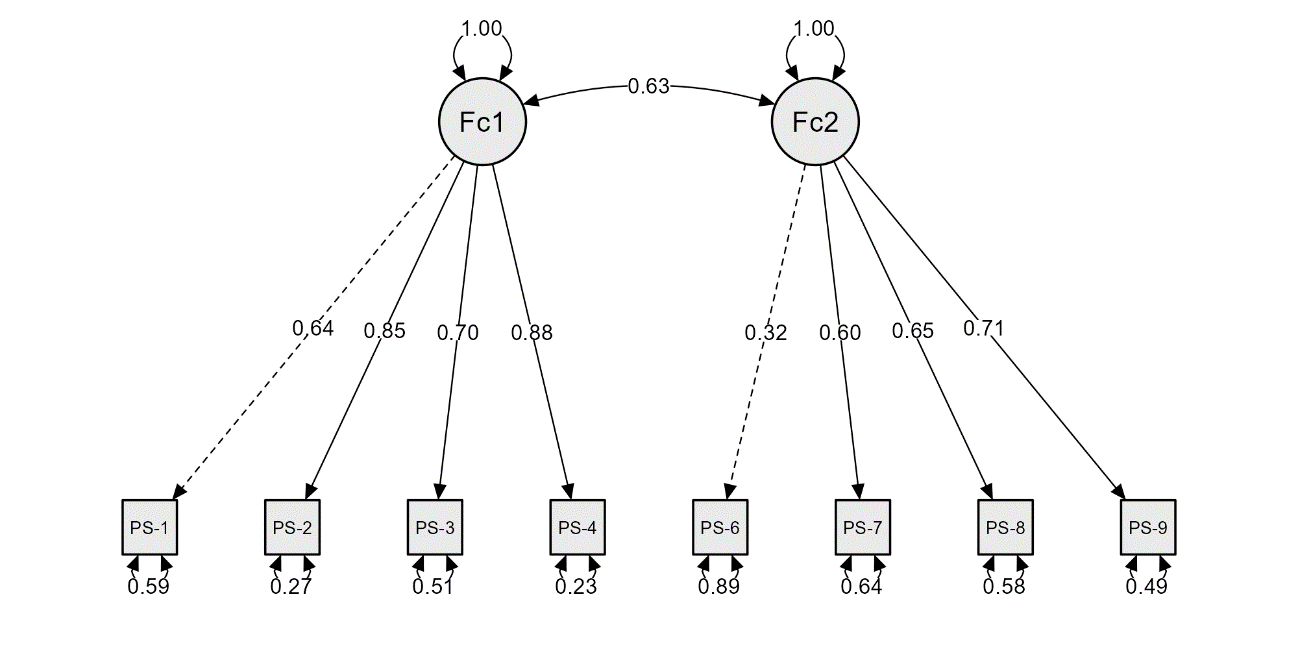


##### 1


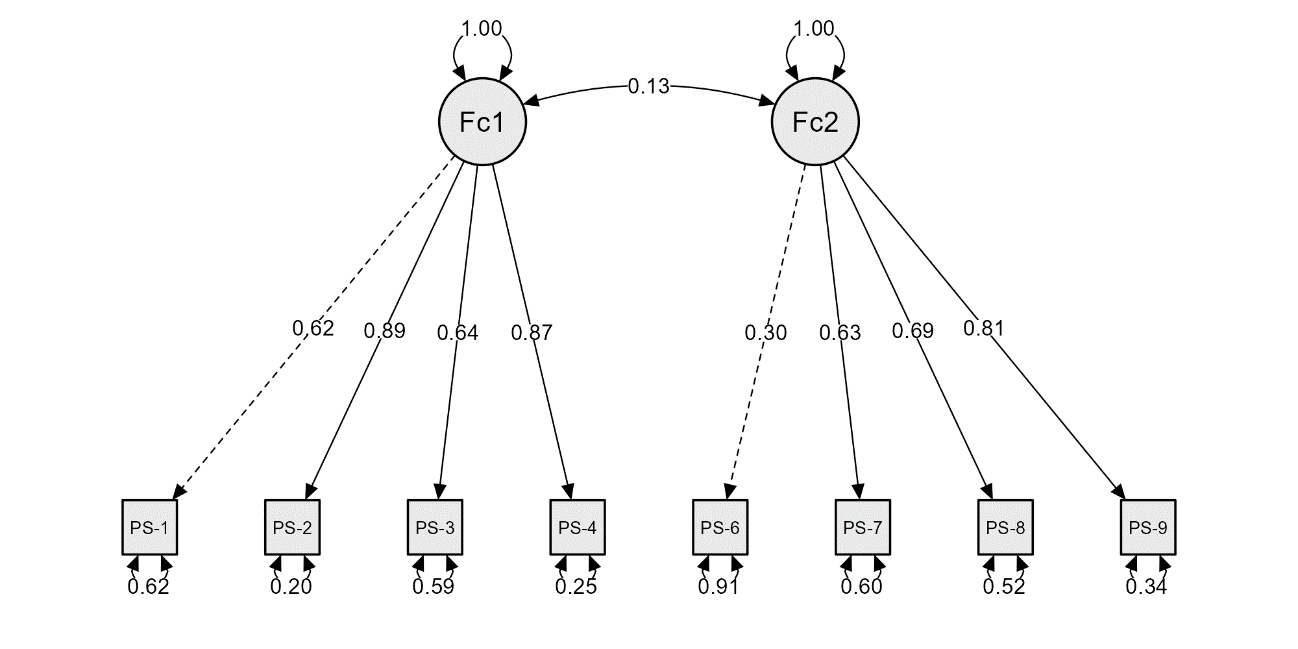


## PS-8 (F1: items 1,2,3,4; F2: items 6,7,8,9)

## Scalar Invariance

| *Chi-square test* | | | | | | | |
| --- | --- | --- | --- | --- | --- | --- | --- |
| Model | | Χ² | | df | | p | |
| Baseline model |  | 1,158.025 |  | 56 |  |  |  |
| Factor model |  | 127.322 |  | 50 |  | < .001 |  |
|  | | | | | | | |
| Note.   The estimator is ML. The test statistic is standard. The standard error method is standard. | | | | | | | |

| *Fit indices* | | | | | | |
| --- | --- | --- | --- | --- | --- | --- |
| Index | | | Value | | | |
| Comparative Fit Index (CFI) |  | | 0.930 | |  | |
| Tucker-Lewis Index (TLI) |  | | 0.921 | |  | |
|  | | | | | | |
| Root mean square error of approximation (RMSEA) | |  | | 0.088 | |  |
| RMSEA 90% CI lower bound | |  | | 0.069 | |  |
| RMSEA 90% CI upper bound | |  | | 0.108 | |  |
| RMSEA p-value | |  | | 7.204×10^-4^ | |  |
| Standardized root mean square residual (SRMR) | |  | | 0.066 | |  |
|  | | | | | | |

| *Factor loadings* | | | | | | | | | | | | | | | | | |
| --- | --- | --- | --- | --- | --- | --- | --- | --- | --- | --- | --- | --- | --- | --- | --- | --- | --- |
|  | | | | | | | | | | | | | | 95% Confidence Interval | | | |
| Group | | Factor | | Indicator | | Estimate | | Std. Error | | z-value | | p | | Lower | | Upper | |
| 2 |  | Factor 1 |  | PS-1 |  | 1.000 |  | 0.000 |  |  |  |  |  | 1.000 |  | 1.000 |  |
|  |  |  |  | PS-2 |  | 1.165 |  | 0.086 |  | 13.548 |  | < .001 |  | 0.996 |  | 1.334 |  |
|  |  |  |  | PS-3 |  | 0.937 |  | 0.081 |  | 11.605 |  | < .001 |  | 0.778 |  | 1.095 |  |
|  |  |  |  | PS-4 |  | 1.180 |  | 0.086 |  | 13.653 |  | < .001 |  | 1.010 |  | 1.349 |  |
|  |  | Factor 2 |  | PS-6 |  | 1.000 |  | 0.000 |  |  |  |  |  | 1.000 |  | 1.000 |  |
|  |  |  |  | PS-7 |  | 1.550 |  | 0.295 |  | 5.252 |  | < .001 |  | 0.972 |  | 2.128 |  |
|  |  |  |  | PS-8 |  | 1.949 |  | 0.367 |  | 5.303 |  | < .001 |  | 1.228 |  | 2.669 |  |
|  |  |  |  | PS-9 |  | 2.217 |  | 0.411 |  | 5.393 |  | < .001 |  | 1.411 |  | 3.023 |  |
| 1 |  | Factor 1 |  | PS-1 |  | 1.000 |  | 0.000 |  |  |  |  |  | 1.000 |  | 1.000 |  |
|  |  |  |  | PS-2 |  | 1.165 |  | 0.086 |  | 13.548 |  | < .001 |  | 0.996 |  | 1.334 |  |
|  |  |  |  | PS-3 |  | 0.937 |  | 0.081 |  | 11.605 |  | < .001 |  | 0.778 |  | 1.095 |  |
|  |  |  |  | PS-4 |  | 1.180 |  | 0.086 |  | 13.653 |  | < .001 |  | 1.010 |  | 1.349 |  |
|  |  | Factor 2 |  | PS-6 |  | 1.000 |  | 0.000 |  |  |  |  |  | 1.000 |  | 1.000 |  |
|  |  |  |  | PS-7 |  | 1.550 |  | 0.295 |  | 5.252 |  | < .001 |  | 0.972 |  | 2.128 |  |
|  |  |  |  | PS-8 |  | 1.949 |  | 0.367 |  | 5.303 |  | < .001 |  | 1.228 |  | 2.669 |  |
|  |  |  |  | PS-9 |  | 2.217 |  | 0.411 |  | 5.393 |  | < .001 |  | 1.411 |  | 3.023 |  |
|  | | | | | | | | | | | | | | | | | |

| *Factor variances* | | | | | | | | | | | | | | | |
| --- | --- | --- | --- | --- | --- | --- | --- | --- | --- | --- | --- | --- | --- | --- | --- |
|  | | | | | | | | | | | | 95% Confidence Interval | | | |
| Group | | Factor | | Estimate | | Std. Error | | z-value | | p | | Lower | | Upper | |
| 2 |  | Factor 1 |  | 0.498 |  | 0.076 |  | 6.548 |  | < .001 |  | 0.349 |  | 0.647 |  |
|  |  | Factor 2 |  | 0.142 |  | 0.051 |  | 2.812 |  | .005 |  | 0.043 |  | 0.242 |  |
| 1 |  | Factor 1 |  | 0.566 |  | 0.125 |  | 4.520 |  | < .001 |  | 0.321 |  | 0.812 |  |
|  |  | Factor 2 |  | 0.171 |  | 0.069 |  | 2.471 |  | .013 |  | 0.035 |  | 0.307 |  |
|  | | | | | | | | | | | | | | | |

| *Factor Covariances* | | | | | | | | | | | | | | | | | | | |
| --- | --- | --- | --- | --- | --- | --- | --- | --- | --- | --- | --- | --- | --- | --- | --- | --- | --- | --- | --- |
|  | | | | | | | | | | | | | | | | 95% Confidence Interval | | | |
| Group | |  | |  | |  | | Estimate | | Std. Error | | z-value | | p | | Lower | | Upper | |
| 2 |  | Factor 1 |  | ↔ |  | Factor 2 |  | 0.167 |  | 0.037 |  | 4.545 |  | < .001 |  | 0.095 |  | 0.239 |  |
| 1 |  | Factor 1 |  | ↔ |  | Factor 2 |  | 0.041 |  | 0.044 |  | 0.948 |  | .343 |  | -0.044 |  | 0.127 |  |
|  | | | | | | | | | | | | | | | | | | | |

| *Residual variances* | | | | | | | | | | | | | | | |
| --- | --- | --- | --- | --- | --- | --- | --- | --- | --- | --- | --- | --- | --- | --- | --- |
|  | | | | | | | | | | | | 95% Confidence Interval | | | |
| Group | | Indicator | | Estimate | | Std. Error | | z-value | | p | | Lower | | Upper | |
| 2 |  | PS-1 |  | 0.704 |  | 0.061 |  | 11.506 |  | < .001 |  | 0.584 |  | 0.824 |  |
|  |  | PS-2 |  | 0.251 |  | 0.032 |  | 7.980 |  | < .001 |  | 0.190 |  | 0.313 |  |
|  |  | PS-3 |  | 0.456 |  | 0.041 |  | 11.089 |  | < .001 |  | 0.375 |  | 0.536 |  |
|  |  | PS-4 |  | 0.213 |  | 0.030 |  | 7.118 |  | < .001 |  | 0.154 |  | 0.271 |  |
|  |  | PS-6 |  | 1.224 |  | 0.101 |  | 12.158 |  | < .001 |  | 1.027 |  | 1.421 |  |
|  |  | PS-7 |  | 0.581 |  | 0.057 |  | 10.180 |  | < .001 |  | 0.469 |  | 0.693 |  |
|  |  | PS-8 |  | 0.792 |  | 0.081 |  | 9.763 |  | < .001 |  | 0.633 |  | 0.951 |  |
|  |  | PS-9 |  | 0.649 |  | 0.080 |  | 8.068 |  | < .001 |  | 0.491 |  | 0.806 |  |
| 1 |  | PS-1 |  | 0.940 |  | 0.163 |  | 5.780 |  | < .001 |  | 0.622 |  | 1.259 |  |
|  |  | PS-2 |  | 0.196 |  | 0.062 |  | 3.187 |  | .001 |  | 0.076 |  | 0.317 |  |
|  |  | PS-3 |  | 0.703 |  | 0.123 |  | 5.705 |  | < .001 |  | 0.462 |  | 0.945 |  |
|  |  | PS-4 |  | 0.267 |  | 0.070 |  | 3.817 |  | < .001 |  | 0.130 |  | 0.403 |  |
|  |  | PS-6 |  | 1.789 |  | 0.295 |  | 6.064 |  | < .001 |  | 1.211 |  | 2.368 |  |
|  |  | PS-7 |  | 0.621 |  | 0.121 |  | 5.138 |  | < .001 |  | 0.384 |  | 0.858 |  |
|  |  | PS-8 |  | 0.829 |  | 0.169 |  | 4.914 |  | < .001 |  | 0.498 |  | 1.160 |  |
|  |  | PS-9 |  | 0.443 |  | 0.140 |  | 3.161 |  | .002 |  | 0.168 |  | 0.718 |  |
|  | | | | | | | | | | | | | | | |

#### Model plots

##### 2


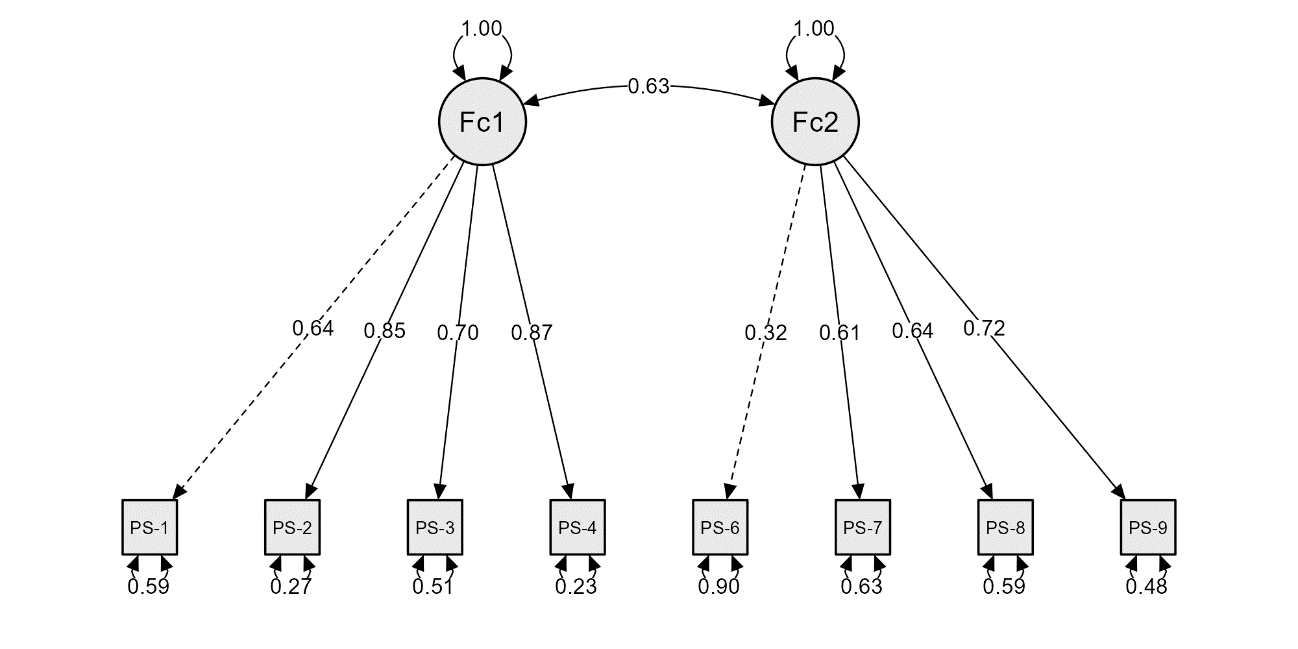


##### 1


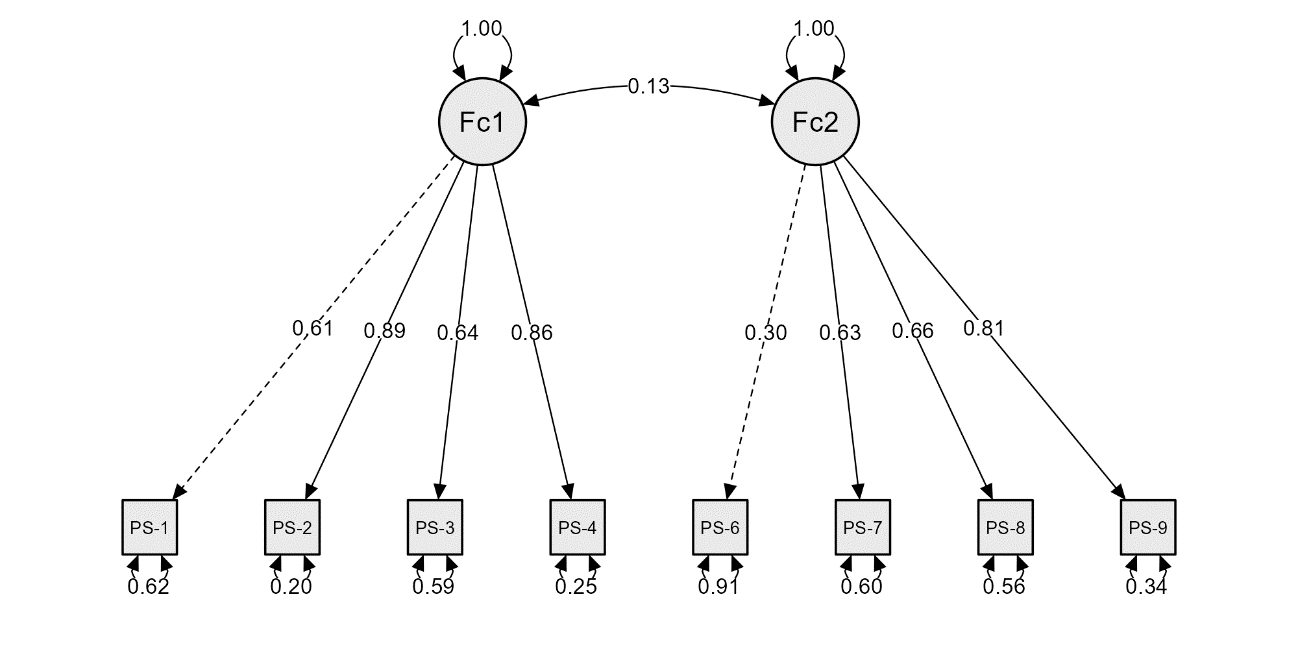


## PS-8 (F1: items 1,2,3,4; F2: items 6,7,8,9)

## Strict Invariance

| *Chi-square test* | | | | | | | |
| --- | --- | --- | --- | --- | --- | --- | --- |
| Model | | Χ² | | df | | p | |
| Baseline model |  | 1,158.025 |  | 56 |  |  |  |
| Factor model |  | 141.810 |  | 58 |  | < .001 |  |
|  | | | | | | | |
| Note.   The estimator is ML. The test statistic is standard. The standard error method is standard. | | | | | | | |

| *Fit indices* | | | | | | |
| --- | --- | --- | --- | --- | --- | --- |
| Index | | | Value | | | |
| Comparative Fit Index (CFI) |  | | 0.924 | |  | |
| Tucker-Lewis Index (TLI) |  | | 0.927 | |  | |
|  | | | | | | |
| Root mean square error of approximation (RMSEA) | |  | | 0.085 | |  |
| RMSEA 90% CI lower bound | |  | | 0.068 | |  |
| RMSEA 90% CI upper bound | |  | | 0.103 | |  |
| RMSEA p-value | |  | | 8.421×10^-4^ | |  |
| Standardized root mean square residual (SRMR) | |  | | 0.066 | |  |
|  | | | | | | |

| *Factor loadings* | | | | | | | | | | | | | | | | | |
| --- | --- | --- | --- | --- | --- | --- | --- | --- | --- | --- | --- | --- | --- | --- | --- | --- | --- |
|  | | | | | | | | | | | | | | 95% Confidence Interval | | | |
| Group | | Factor | | Indicator | | Estimate | | Std. Error | | z-value | | p | | Lower | | Upper | |
| 2 |  | Factor 1 |  | PS-1 |  | 1.000 |  | 0.000 |  |  |  |  |  | 1.000 |  | 1.000 |  |
|  |  |  |  | PS-2 |  | 1.183 |  | 0.089 |  | 13.339 |  | < .001 |  | 1.009 |  | 1.356 |  |
|  |  |  |  | PS-3 |  | 0.935 |  | 0.083 |  | 11.298 |  | < .001 |  | 0.773 |  | 1.098 |  |
|  |  |  |  | PS-4 |  | 1.199 |  | 0.089 |  | 13.396 |  | < .001 |  | 1.023 |  | 1.374 |  |
|  |  | Factor 2 |  | PS-6 |  | 1.000 |  | 0.000 |  |  |  |  |  | 1.000 |  | 1.000 |  |
|  |  |  |  | PS-7 |  | 1.571 |  | 0.302 |  | 5.199 |  | < .001 |  | 0.979 |  | 2.164 |  |
|  |  |  |  | PS-8 |  | 1.960 |  | 0.374 |  | 5.239 |  | < .001 |  | 1.227 |  | 2.694 |  |
|  |  |  |  | PS-9 |  | 2.198 |  | 0.412 |  | 5.330 |  | < .001 |  | 1.390 |  | 3.007 |  |
| 1 |  | Factor 1 |  | PS-1 |  | 1.000 |  | 0.000 |  |  |  |  |  | 1.000 |  | 1.000 |  |
|  |  |  |  | PS-2 |  | 1.183 |  | 0.089 |  | 13.339 |  | < .001 |  | 1.009 |  | 1.356 |  |
|  |  |  |  | PS-3 |  | 0.935 |  | 0.083 |  | 11.298 |  | < .001 |  | 0.773 |  | 1.098 |  |
|  |  |  |  | PS-4 |  | 1.199 |  | 0.089 |  | 13.396 |  | < .001 |  | 1.023 |  | 1.374 |  |
|  |  | Factor 2 |  | PS-6 |  | 1.000 |  | 0.000 |  |  |  |  |  | 1.000 |  | 1.000 |  |
|  |  |  |  | PS-7 |  | 1.571 |  | 0.302 |  | 5.199 |  | < .001 |  | 0.979 |  | 2.164 |  |
|  |  |  |  | PS-8 |  | 1.960 |  | 0.374 |  | 5.239 |  | < .001 |  | 1.227 |  | 2.694 |  |
|  |  |  |  | PS-9 |  | 2.198 |  | 0.412 |  | 5.330 |  | < .001 |  | 1.390 |  | 3.007 |  |
|  | | | | | | | | | | | | | | | | | |

| *Factor variances* | | | | | | | | | | | | | | | |
| --- | --- | --- | --- | --- | --- | --- | --- | --- | --- | --- | --- | --- | --- | --- | --- |
|  | | | | | | | | | | | | 95% Confidence Interval | | | |
| Group | | Factor | | Estimate | | Std. Error | | z-value | | p | | Lower | | Upper | |
| 2 |  | Factor 1 |  | 0.484 |  | 0.075 |  | 6.427 |  | < .001 |  | 0.336 |  | 0.631 |  |
|  |  | Factor 2 |  | 0.142 |  | 0.051 |  | 2.772 |  | .006 |  | 0.042 |  | 0.243 |  |
| 1 |  | Factor 1 |  | 0.540 |  | 0.119 |  | 4.528 |  | < .001 |  | 0.306 |  | 0.774 |  |
|  |  | Factor 2 |  | 0.162 |  | 0.066 |  | 2.458 |  | .014 |  | 0.033 |  | 0.291 |  |
|  | | | | | | | | | | | | | | | |

| *Factor Covariances* | | | | | | | | | | | | | | | | | | | |
| --- | --- | --- | --- | --- | --- | --- | --- | --- | --- | --- | --- | --- | --- | --- | --- | --- | --- | --- | --- |
|  | | | | | | | | | | | | | | | | 95% Confidence Interval | | | |
| Group | |  | |  | |  | | Estimate | | Std. Error | | z-value | | p | | Lower | | Upper | |
| 2 |  | Factor 1 |  | ↔ |  | Factor 2 |  | 0.164 |  | 0.037 |  | 4.489 |  | < .001 |  | 0.093 |  | 0.236 |  |
| 1 |  | Factor 1 |  | ↔ |  | Factor 2 |  | 0.051 |  | 0.043 |  | 1.178 |  | .239 |  | -0.034 |  | 0.135 |  |
|  | | | | | | | | | | | | | | | | | | | |

| *Residual variances* | | | | | | | | | | | | | | | |
| --- | --- | --- | --- | --- | --- | --- | --- | --- | --- | --- | --- | --- | --- | --- | --- |
|  | | | | | | | | | | | | 95% Confidence Interval | | | |
| Group | | Indicator | | Estimate | | Std. Error | | z-value | | p | | Lower | | Upper | |
| 2 |  | PS-1 |  | 0.752 |  | 0.058 |  | 12.870 |  | < .001 |  | 0.637 |  | 0.866 |  |
|  |  | PS-2 |  | 0.238 |  | 0.029 |  | 8.184 |  | < .001 |  | 0.181 |  | 0.295 |  |
|  |  | PS-3 |  | 0.509 |  | 0.041 |  | 12.507 |  | < .001 |  | 0.429 |  | 0.589 |  |
|  |  | PS-4 |  | 0.224 |  | 0.029 |  | 7.754 |  | < .001 |  | 0.168 |  | 0.281 |  |
|  |  | PS-6 |  | 1.328 |  | 0.098 |  | 13.550 |  | < .001 |  | 1.136 |  | 1.520 |  |
|  |  | PS-7 |  | 0.584 |  | 0.053 |  | 11.013 |  | < .001 |  | 0.480 |  | 0.688 |  |
|  |  | PS-8 |  | 0.801 |  | 0.076 |  | 10.573 |  | < .001 |  | 0.652 |  | 0.949 |  |
|  |  | PS-9 |  | 0.625 |  | 0.074 |  | 8.488 |  | < .001 |  | 0.481 |  | 0.770 |  |
| 1 |  | PS-1 |  | 0.752 |  | 0.058 |  | 12.870 |  | < .001 |  | 0.637 |  | 0.866 |  |
|  |  | PS-2 |  | 0.238 |  | 0.029 |  | 8.184 |  | < .001 |  | 0.181 |  | 0.295 |  |
|  |  | PS-3 |  | 0.509 |  | 0.041 |  | 12.507 |  | < .001 |  | 0.429 |  | 0.589 |  |
|  |  | PS-4 |  | 0.224 |  | 0.029 |  | 7.754 |  | < .001 |  | 0.168 |  | 0.281 |  |
|  |  | PS-6 |  | 1.328 |  | 0.098 |  | 13.550 |  | < .001 |  | 1.136 |  | 1.520 |  |
|  |  | PS-7 |  | 0.584 |  | 0.053 |  | 11.013 |  | < .001 |  | 0.480 |  | 0.688 |  |
|  |  | PS-8 |  | 0.801 |  | 0.076 |  | 10.573 |  | < .001 |  | 0.652 |  | 0.949 |  |
|  |  | PS-9 |  | 0.625 |  | 0.074 |  | 8.488 |  | < .001 |  | 0.481 |  | 0.770 |  |
|  | | | | | | | | | | | | | | | |

#### Model plots

##### 2


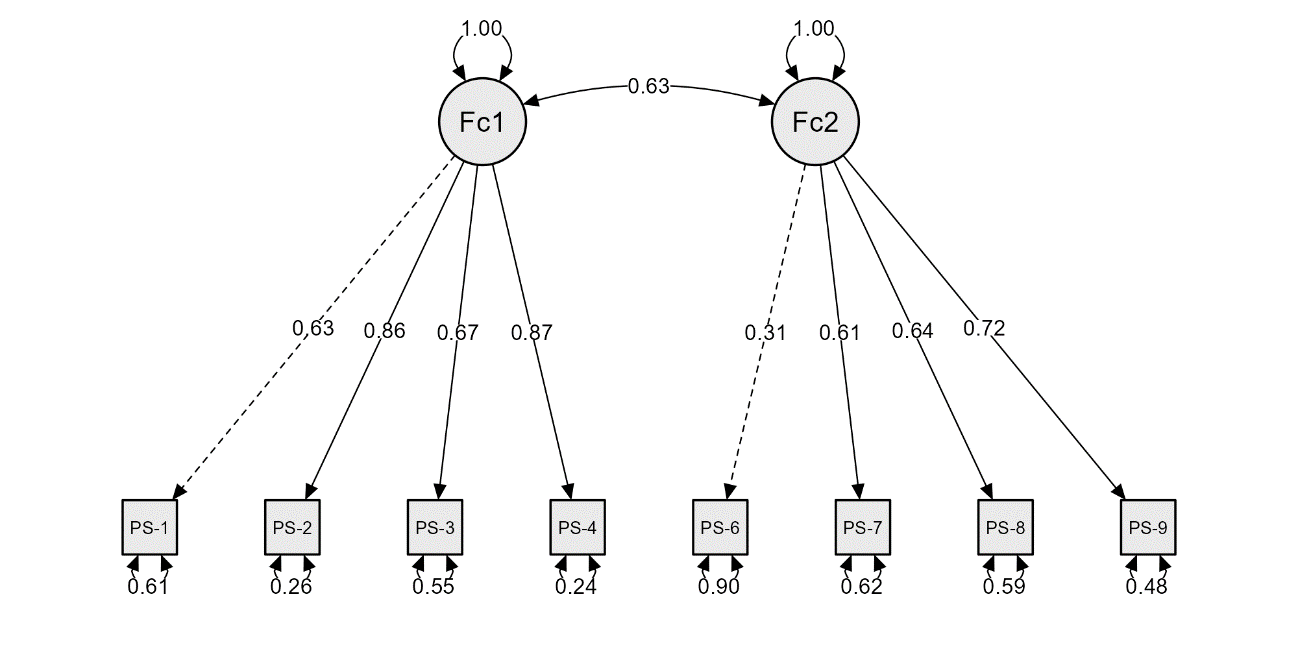


##### 1


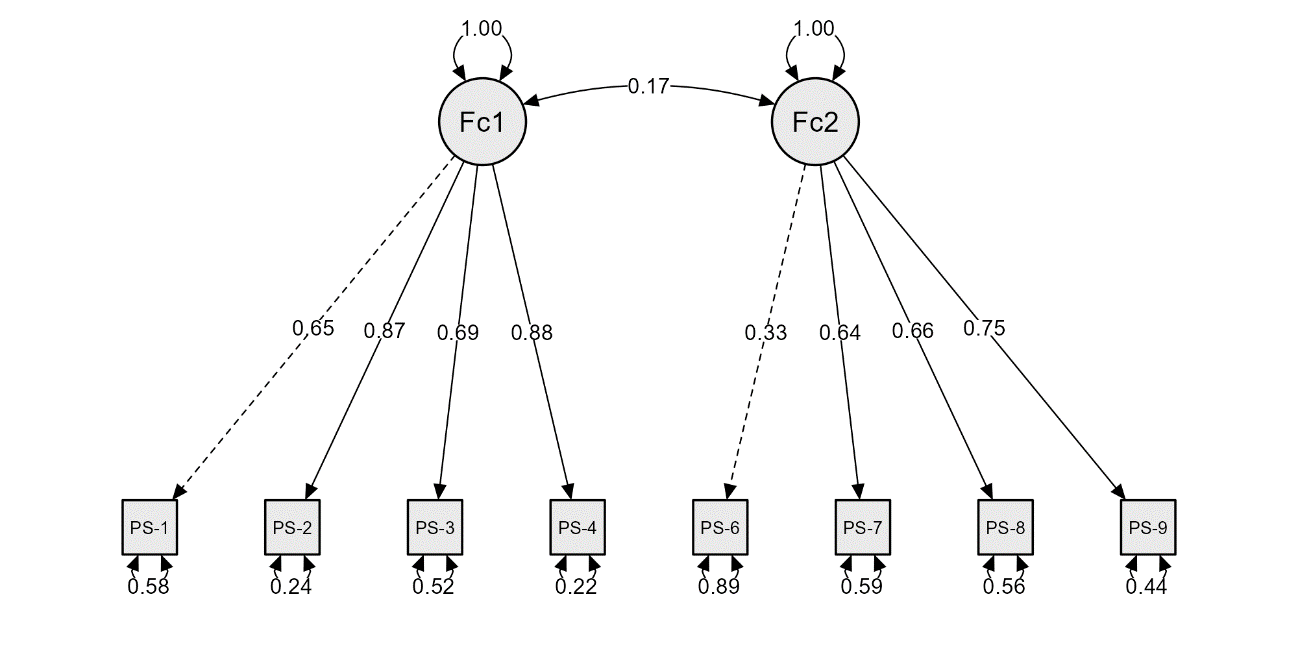

Supplement: Supplementary file 1 [file SupplementaryFile1.docx]
